# Co-activation of super-enhancer-driven CCAT1 by TP63 and SOX2 promotes squamous cancer progression

**DOI:** 10.1038/s41467-018-06081-9

**Published:** 2018-09-06

**Authors:** Yuan Jiang, Yan-Yi Jiang, Jian-Jun Xie, Anand Mayakonda, Masaharu Hazawa, Li Chen, Jin-Fen Xiao, Chun-Quan Li, Mo-Li Huang, Ling-Wen Ding, Qiao-Yang Sun, Liang Xu, Deepika Kanojia, Maya Jeitany, Jian-Wen Deng, Lian-Di Liao, Harmik J. Soukiasian, Benjamin P. Berman, Jia-Jie Hao, Li-Yan Xu, En-Min Li, Ming-Rong Wang, Xin-Gang Bi, De-Chen Lin, H. Phillip Koeffler

**Affiliations:** 1 Cancer Science Institute of Singapore, National University of Singapore, Singapore, 117599 Singapore; 2 Department of Biochemistry and Molecular Biology, Medical College of Shantou University, Shantou, 515041 China; 3 Cell-Bionomics Research Unit, Innovative Integrated Bio-Research Core, Institute for Frontier Science Initiative, Kanazawa University, Kanazawa, 920-1192 Ishikawa Japan; 4 Department of Medicine, Cedars-Sinai Medical Center, Los Angeles, 90048 USA; 5 School of Medical Informatics, Daqing Campus, Harbin Medical University, Daqing, 163319 China; 6 Institute of Oncologic Pathology, Medical College of Shantou University, Shantou, 515041 China; 7 Department of Surgery, Cedars-Sinai Medical Center, Los Angeles, 90048 CA USA; 8 Center for Bioinformatics and Functional Genomics, Cedars-Sinai Medical Center, Los Angeles, 90048 CA USA; 9 State Key Laboratory of Molecular Oncology, National Cancer Center/National Clinical Research Center for Cancer/Cancer Hospital, Chinese Academy of Medical Sciences and Peking Union Medical College, Beijing, 100021 China; 10 Department of Urology, National Cancer Center/National Clinical Research Center for Cancer/Cancer Hospital, Chinese Academy of Medical Sciences and Peking Union Medical College, Beijing, 100021 China; 11 National University Cancer Institute, National University Hospital, Singapore, 119074 Singapore

## Abstract

Squamous cell carcinomas (SCCs) are aggressive malignancies. Previous report demonstrated that master transcription factors (TFs) TP63 and SOX2 exhibited overlapping genomic occupancy in SCCs. However, functional consequence of their frequent co-localization at super-enhancers remains incompletely understood. Here, epigenomic profilings of different types of SCCs reveal that TP63 and SOX2 cooperatively and lineage-specifically regulate long non-coding RNA (lncRNA) *CCAT1* expression, through activation of its super-enhancers and promoter. Silencing of CCAT1 substantially reduces cellular growth both in vitro and in vivo, phenotyping the effect of inhibiting either TP63 or SOX2. ChIRP analysis shows that CCAT1 forms a complex with TP63 and SOX2, which regulates EGFR expression by binding to the super-enhancers of *EGFR*, thereby activating both MEK/ERK1/2 and PI3K/AKT signaling pathways. These results together identify a SCC-specific DNA/RNA/protein complex which activates TP63/SOX2-CCAT1-EGFR cascade and promotes SCC tumorigenesis, advancing our understanding of transcription dysregulation in cancer biology mediated by master TFs and super-enhancers.

## Abstract (web summary)

Master regulator transcription factors TP63 and SOX2 have been reported to overlap in genomic occupancy in squamous cell carcinomas (SCCs). Here, the authors demonstrate that TP63 and SOX2 promote co-operatively long non-coding RNA CCAT1 expression through activating its super-enhancer, and CCAT1 forms a complex with TP63 and SOX2, which regulates EGFR super-enhancers and enhances both the MEK/ERK1/2 and PI3K/AKT signaling pathways in SCC.

## Introduction

Squamous cell carcinomas (SCCs) are aggressive malignancies arising from squamous epithelium of various organs, such as esophagus, head and neck, lung and skin. Recently, large-scale molecular studies have characterized comprehensively both genomic and epigenomic (predominantly at the methylation level) alterations in different forms of SCCs^1–6^. However, these molecular profilings have not substantially improved clinical management of SCC patients, and no effective targeted regimens have been established for these cancers.

Although derived from diverse epithelial origins, SCCs have a number of unified genomic characteristics, with some being lineage-specific. The most notable SCC-specific genomic lesions target several transcription factors (TFs) with prominent functions in both healthy and neoplastic squamous cells, including *SOX2, TP63, ZNF750,* and NOTCH family genes. Specifically, *SOX2* and *TP63* are frequently co-amplified and overexpressed^1,2,5^, while the NOTCH family genes and *ZNF750* exhibit recurrent loss-of-function mutations in SCCs^4,5,7,8^. These genetic alterations rarely occur in non-SCC cancers, highlighting their pathogenic significance in SCC biology.

Super-enhancers play prominent roles in driving expression of cell-type-specific genes through interacting with master TFs, co-factors, RNA polymerase II as well as non-coding RNAs^9–12^. We recently identified and characterized super-enhancer-associated genes with lineage-specific expression patterns in esophageal SCC (ESCC)^13,14^, including *TP63* and *SOX2*. Previous investigations have shown that deletion of *TP63* causes striking defects in epidermal development, highlighting its key role in the regulation of squamous cell differentiation and proliferation^15–17^. In squamous cancer cells, overexpression of either SOX2 or TP63 promotes proliferation and tumorigenesis^1,18–21^, suggesting oncogenic functions of these master TFs. Notably, Watanabe et al. (2014) showed that genomic occupancy of TP63 and SOX2 were significantly overlapping, and suggested functional co-operation between these two TFs in SCCs^22^. However, whether and how super-enhancers are under regulation by TP63 and SOX2, and its associated biological significance in SCCs remain unexplored.

Here, we perform epigenomic profilings to characterize the super-enhancer landscape in SCCs and investigate the importance of co-localization of TP63 and SOX2 at super-enhancer regions. Integrative analysis shows that TP63 and SOX2 co-bind to the promoter and super-enhancer regions of a long non-coding RNA (lncRNA), *CCAT1*, a novel SCC oncogenic lncRNA. Further mechanistic exploration demonstrates a complex interplay between CCAT1, TP63, and SOX2 in the transcriptional activation of EGFR, resulting in the hyper-activation of EGFR downstream pathways in SCC cells.

## Results

### Super-enhancer regions are co-occupied by TP63 and SOX2 in SCC

**Fig. 1:**
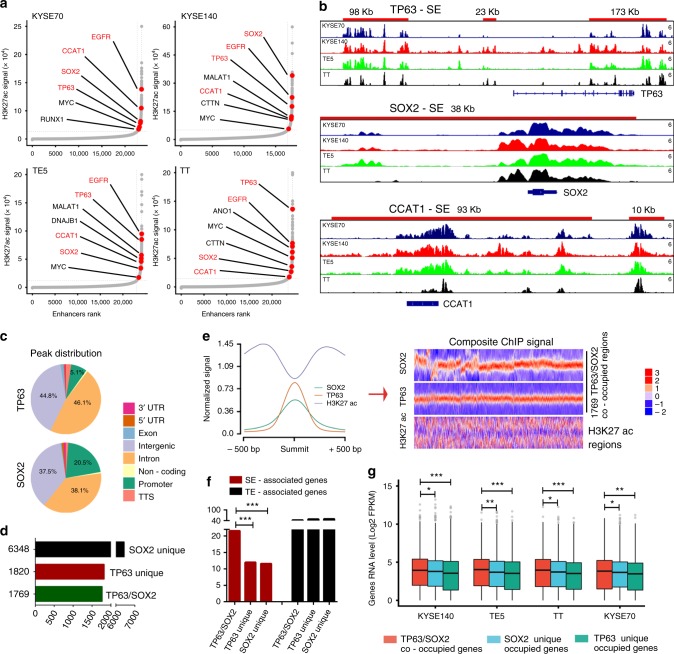
Super-enhancers are associated with TP63 and SOX2 in SCCs. **a** Hockey stick plots on the basis of their input-normalized H3K27ac signals in KYSE70, KYSE140, TE5, and TT cell lines. Know super-enhancer-associated oncogenes are highlighted. **b** Gene tracks of H3K27ac ChIP-seq occupancy at representative super-enhancer-associated gene loci in four cell lines. *X* axis shows genomic position and *Y* axis shows signal of ChIP-seq occupancy in units of reads per million mapped reads per base pair (rpm/bp). **c** Genome-wide distribution of TP63 and SOX2 ChIP-seq peaks in TE5 cells. **d** The number of TP63 unique, SOX2 unique and TP63/SOX2 co-occupied genomic loci in TE5 cells. **e** Left: line plots showing ChIP-seq signals of TP63, SOX2 and H3K27ac centered at the summit of TP63 and SOX2 peaks in TE5 cells. Right: heatmap of ChIP-seq signals for TP63, SOX2, and H3K27ac (±500 bp windows around the center of summit) rank ordered by TP63 signal. Red reflects enrichment. **f** Proportion of super-enhancer (SE)-associated genes (red) and typical-enhancer (TE)-associated genes (black) either uniquely bound or co-bound by TP63 and SOX2. **g** mRNA level of super-enhancer-associated genes either uniquely bound or co-bound by TP63 and SOX2. Red, blue, and green box plot represents the expression levels of TP63/SOX2 co-occupied genes, TP63 uniquely occupied genes and SOX2 uniquely occupied genes, respectively

Cancer cells acquire super-enhancers to drive expression of prominent oncogenes by recruiting a high density of TFs, coactivators, and noncoding RNAs^9,12,23,24^. To explore whether and how super-enhancers are regulated by SCC master TFs (TP63 and SOX2), we first performed chromatin immunoprecipitation sequencing (ChIP-seq) with antibodies against H3K27 acetylation (H3K27ac), TP63, and SOX2. Super-enhancer-associated genes were annotated (Fig. 1a, b and Supplementary Data 1-4). Consistent with our previous report, a numbered of well-defined SCC oncogenes had super-enhancers, (e.g. *TP63*, *SOX2*, *EGFR,* and *MYC*)^13,20–22,25^ in four ESCC cell lines. SCC-specific expression pattern was, as expected, observed with both TP63 and SOX2 (Supplementary Fig. 1). A direct interaction between TP63 and SOX2 in SCC was also verified by our immunoprecipitation (IP) results (Supplementary Fig. 2), confirming a previous report^22^.

Not surprisingly, the majority of TP63 and SOX2 ChIP-seq peaks were located at intergenic and intron regions (Fig. 1c and Supplementary Fig. 3). Importantly, almost half of TP63 binding peaks overlapped with SOX2 enriched loci (Fig. 1d, e). However, in embryonic stem cells, such overlapping genomic pattern of TP63 and SOX2 was absent^22^, suggesting their unique functional interplay in SCCs. We observed prominently enriched H3K27ac signals adjacent to both TP63 and SOX2 peaks (Fig. 1e), suggesting that transcriptional activation was associated with the binding of these two TFs.

To gain additional insights into the interactions between super-enhancers and TP63 and SOX2, we assigned TP63 and SOX2 ChIP-seq peaks to both super-enhancers and typical-enhancers. Notably, the co-occupancy of TP63/SOX2 was significantly enriched in super-enhancer-associated genes, relative to unique occupancy from either TFs (Fig. 1f left three columns). This significant enrichment of co-binding was specific to super-enhancer elements, as it was not observed in typical-enhancers (Fig. 1f right three columns). Moreover, super-enhancer-associated genes which were co-bound by TP63 and SOX2 consistently had higher mRNA levels relative to those unique-bound by either TP63 or SOX2 across all four SCC cell lines (Fig. 1g). These results together characterized the landscape of ESCC super-enhancers, and suggest that TP63 or SOX2 co-operatively activate a subset of these super-enhancers with a higher potency.

### Identification of CCAT1 as a key target co-regulated by TP63 and SOX2 through a super-enhancer

**Fig. 2:**
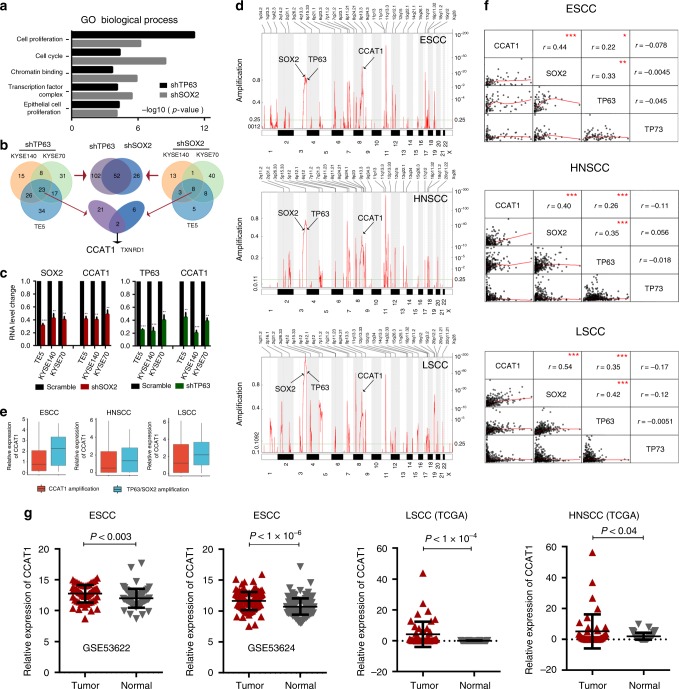
CCAT1 is overexpressed and regulated by TP63 and SOX2 in SCCs. **a** GO functional categories of downregulated genes upon TP63 or SOX2 silencing. **b** Integrative analysis of TP63 and SOX2 co-regulated downstream super-enhancer targets, which are co-occupied by TP63 and SOX2 in three SCC cell lines. **c** CCAT1 mRNA expression upon silencing of either TP63 or SOX2. Data are presented as mean ± SD of three replicates. ** *P* < 0.01, *** *P* < 0.001. *P* values were determined using *t*-test. **d** Plots showing high-level amplifications of *TP63*, *SOX2*, and *CCAT1* loci in esophageal squamous cell carcinomas (ESCC), head and neck squamous cell carcinomas (HNSCC) and lung squamous cell carcinomas (LSCC). *X* axis shows chromosomal regions, *Y* axis shows the G score (left) and false discovery rate *q*-values (right). **e** Relative expression of CCAT1 in *CCAT1* independently amplified and *TP63*/*SOX2* co-amplified tissues. Red or blue box plot represent CCAT1 or TP63/SOX2 co-amplification samples, respectively. **f** Correlation plots comparing CCAT1, SOX2, and TP63 expression in ESCC, HNSCC, and LSCC. *TP73* was included as a negative control. *r* represents correlation value; *represents *P* values. * *P* < 0.05, ** *P* < 0.01, *** *P* < 0.001. *P* values were determined using *t*-test. Data of **d**–**f** from the Cancer Genome Atlas Network (TCGA). **g** CCAT1 expression in tumor and nonmalignant samples across three types of SCCs. Data from GSE53622, GSE53624, and TCGA. Significance was marked with *P* values

To identify gene targets which are regulated by TP63/SOX2, we silenced either TP63 or SOX2 with shRNAs in SCC cell lines (Supplementary Fig. 4) and followed by whole-transcriptome sequencing (RNA-seq) (Fig. 2a, b and Supplementary Data 5-8). Given our earlier findings that TP63- and SOX2-occupied regions were strongly enriched for H3K27ac modification, we primarily focused on genes that were downregulated following the silencing of these two TFs. Gene Ontology (GO) analysis showed that downregulated genes (decreased greater than onefold relative to control) upon TP63 or SOX2 silencing were strongly enriched for cellular phenotypes important for cancer biology, including cell-cycle regulation, chromatin binding, and cell proliferation (Fig. 2a). To identify high-confident downstream targets co-regulated by TP63/SOX2, we further required that these transcripts were co-occupied by the two TFs based on our ChIP-seq data. As a result, we identified a total of 154 and 78 downregulated super-enhancer-associated transcripts upon knockdown of either TP63 or SOX2, respectively. Importantly, 52 of these transcripts were overlapped (*P* < 10^−10^, two-tailed Student *t* tests), suggesting a strong co-regulation of the down-stream targets between TP63 and SOX2. Among these 52 transcripts, *ETV4* was experimentally validated as a common target of the two TFs^22^. Notably, two transcripts (*CCAT1* and *TXNRD1*) consistently had reduced mRNA levels upon either TP63 or SOX2 knockdown (Fig. 2b) across all three ESCC cell lines.

*CCAT1* (Colon cancer associated transcript-1) is a lncRNA, which was initially noted to be highly expressed in colon cancers^26^. The oncogenic property of CCAT1 was recently reported in several types of tumors, including cancers of the liver, gallbladder and stomach, as well as ESCC^27–30^. Notably, we also identified *CCAT1* as a super-enhancer-associated gene, which was strongly expressed in ESCC (Fig. 1a). We validated that either TP63 or SOX2 knockdown caused significant reduction of the mRNA level of CCAT1, while overexpression either of TFs induced CCAT1 expression in SCC cell lines (Fig. 2c and Supplementary Fig. 5). However, expression of TP63 or SOX2 was not altered following silencing of CCAT1 (Supplementary Fig. 6), suggesting that CCAT1 is the downstream target of TP63 and SOX2 in SCCs.

Human *CCAT1* gene is located at chr8q24.21, a recurrently amplified genomic region in SCCs. Therefore, we analyzed copy number alteration of *CCAT1* in three major SCC types, ESCC, head and neck SCC (HNSCC) and lung SCC (LSCC), based on the data from The Cancer Genome Atlas Network (TCGA). As expected, in all SCC types, recurrent co-amplification of *TP63* and *SOX2* was detected (Fig. 2d). Importantly, although *CCAT1* also exhibited genomic amplification (Fig. 2d), the level of CCAT1 mRNA was markedly higher in *TP63*/*SOX2* co-amplified samples relative to *CCAT1*-amplified samples across different types of SCC tumors (Fig. 2e), suggesting that TP63/SOX2 co-regulation plays a more important role in driving CCAT1 expression than the amplification of *CCAT1* DNA itself. Furthermore, the mRNA level of CCAT1 correlated significantly with that of TP63 and SOX2 in all SCC tumors (Fig. 2f). Given that TP63 and SOX2 are often overexpressed in SCC tumors compared with corresponding nonmalignant tissues, we next analyzed CCAT1 expression levels. Indeed, mRNA upregulation of CCAT1 in tumor samples relative to nonmalignant tissues was consistently observed in all types of SCCs (Fig. 2g). Together, these results identify super-enhancer-associated CCAT1 as a downstream target co-regulated by TP63 and SOX2, which are recurrently co-amplified in SCCs.

### CCAT1 promotes SCC cell proliferation both in vitro and in vivo

**Fig. 3:**
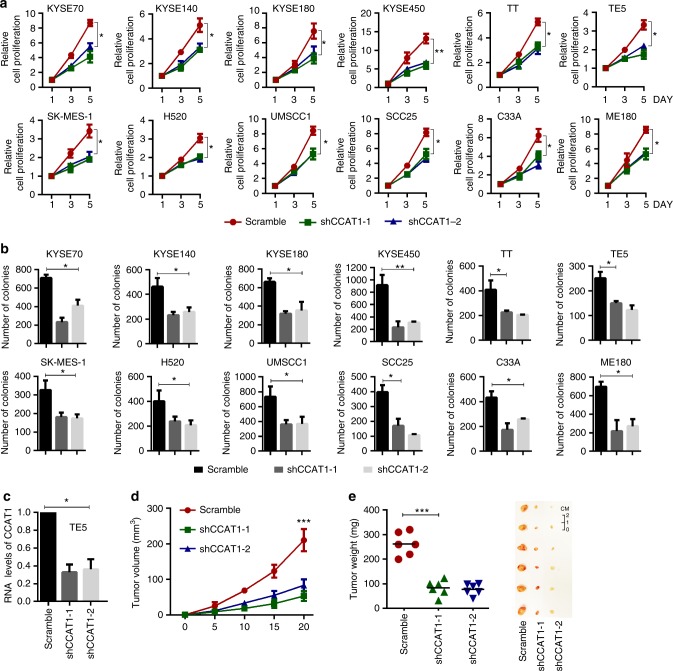
CCAT1 promotes SCC cell proliferation both in vitro and in vivo. **a** Cell viability and **b** Colony formation assays of SCC cell lines upon CCAT1 knockdown with two different shRNA targets. **c** Relative expression of CCAT1 following silencing of CCAT1 in TE5 cells. Bars of **a**–**c** represent mean ± SD of three experimental replicates. * *P* < 0.05, ** *P* < 0.01. *P* values were determined using *t*-test. **d** Tumor volumes were measured at the indicated time points in scramble and CCAT1 knockdown xenograft mice. **e** Left: summary of mean tumor weight measured at end point; Right: images of dissected tumors. *N* = 6. ***P < 0.001. *P* values were determined using *t*-test

To begin to probe the biological function of CCAT1 in SCCs, we procured a total of 28 SCC cell lines of ESCC, LSCC, HNSCC and cervical SCC (CSCC) (Supplementary Fig. 7a). A total of 12 of these cell lines with high CCAT1 expression representing each type of SCC were selected for loss-of-function assays (Supplementary Fig. 7a, b). Importantly, silencing of CCAT1 strongly impaired both cell viability and clonogenic capacity in all 12 SCC lines (Fig. 3a, b). We next ectopically overexpressed CCAT1 in both KYSE150 and KYSE510 cells, which had the lowest level of CCAT1 among all 13 lines (Supplementary Fig. 8a-c). Importantly, overexpression of CCAT1 significantly increased both the proliferation and colony growth of these two cell lines (Supplementary Fig. 8d, e). Moreover, CCAT1 knockdown in the xenograft assays resulted in marked reduction in both volume and mass of the tumors (Fig. 3c–e). These data showed a strong oncogenic potential of CCAT1 in SCC cells.

### TP63 and SOX2 co-occupy at both the promoter and super-enhancers of *CCAT1*

**Fig. 4:**
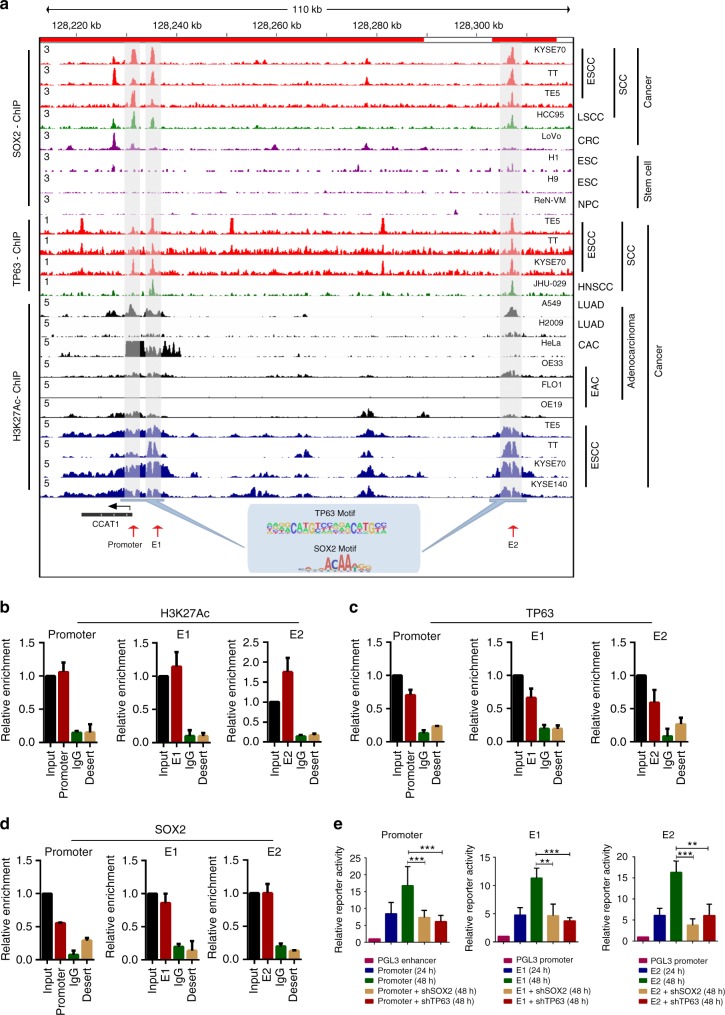
TP63 and SOX2 directly co-regulate CCAT1 transcription. **a** Occupancy profiles of TP63, SOX2, and H3K27ac at the *CCAT1* promoter and super-enhancer regions in various types of cells, including SCCs (ESCC, LSCC, and HNSCC), lung adenocarcinoma (LAC), cervical adenocarcinoma (CAC), esophageal adenocarcinoma (EAC), and embryonic stem cells (ESC). Grey shadings indicate the co-occupancy of TP63, SOX2, and H3K27ac. Blue shadings show TP63 and SOX2 motifs. Except for ChIP-seq, results of ESCC and EAC cells were generated in house, other ChIP-seq profiles were re-analyzed based on Cistrome Data Browser (http://cistrome.org/db/#/). **b**–**d** ChIP-qPCR analysis for enrichment of H3K27ac (**b**), TP63 (**c**) and SOX2 (**d**) at the promoter and super-enhancers (divided into enhancer 1, E1 and enhancer 2, E2) identified in **a**. Shown are the means of technical triplicates in a representative experiment, performed twice. Error bars indicate mean ± SD from three biological replicates per group. IgG and desert represent negative controls of antibody and gene desert region, respectively. **e** *CCAT1* promoter and enhancer activities are measured by luciferase reporter assays at indicated times. Luciferase activity is reduced by TP63 or SOX2 knockdown in TE5 cells. Data are mean ± SD from three biological replicates per group. CRC: colorectal cancer, NPC: neural progenitor cell

To explore how CCAT1 was regulated by TP63 and SOX2, we analyzed ChIP-seq data of H3K27ac, TP63, and SOX2 generated from SCC cells, and compared them to those from other cells types, including embryonic stem cells and adenocarcinoma cells from various organs. The first observation was that the super-enhancers flanking *CCAT1* (denoted by two red bars on top of Fig. 4a) were SCC-specific, since they were either undetectable or much weaker in adenocarcinoma cells of the esophagus (OE33, OE19, Flo-1), lung (A549 and H2009), and cervix (HeLa). Importantly, both *CCAT1* promoter and super-enhancer regions [particularly constituents Enhancer 1 (E1) and Enhancer 2 (E2)] were co-occupied by TP63 and SOX2 in almost all SCC cell lines (except for TP63 at the promoter of JHU-029 cells, Fig. 4a). Again, this binding pattern was absent in non-SCC cells, suggesting the lineage-specific feature.

To validate the ChIP-seq results, ChIP-qPCR was performed to quantify the occupancy of H3K27ac, TP63, and SOX2, and their enrichment was confirmed at the promoter and super-enhancers (E1 and E2) of *CCAT1* (Fig. 4b–d). We further performed luciferase reporter assays, and demonstrated that the reporter activity was prominently increased upon transfection of either *CCAT1* promoter or enhancer (E1 or E2) (Fig. 4e and Supplementary Fig. 9). Importantly, silencing of TP63 or SOX2 potently inhibited this reporter activity (Fig. 4e and Supplementary Fig. 9). These data demonstrate that TP63 and SOX2 co-occupy the promoter and super-enhancers of *CCAT1*, thereby activating its transcription in SCC cells in a lineage-specific manner.

### CCAT1 activates both MEK/ERK1/2 and PI3K/AKT signaling pathways

**Fig. 5:**
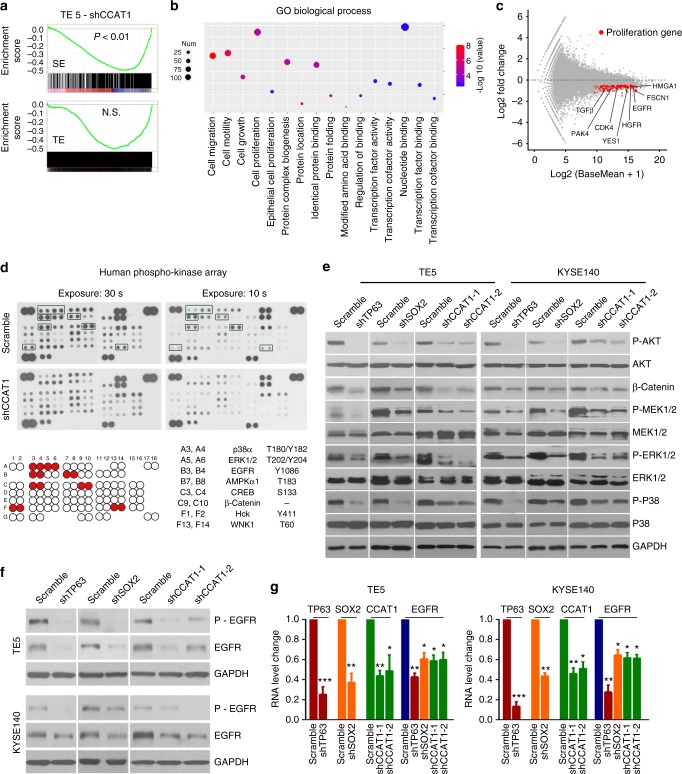
CCAT1 regulates MEK/ERK1/2 and PI3K/AKT signaling pathways. **a** Gene set enrichment analysis (GSEA) of fold changes of either super-enhancer (SE)-or typical-enhancer (TE)-associated transcripts following CCAT1 silencing. **b** GO enrichment analysis of downregulated genes upon CCAT1 knockdown. Color of circles denotes fold changes and dot size represents the number (num) of genes enriched. **c** MA plot analysis of RNA-seq showing differentially expressed genes upon CCAT1 silencing. Red dots are representative downregulated proliferation genes. **d** Human phospho-kinase array detects phosphorylated proteins in scramble and CCAT1-silenced TE5 cells. Red dots represent phosphorylated proteins showing alterations, and these proteins and their phosphorylation sites were shown on bottom right. **e** Western blotting analysis of the proteins highlighted in **d** and mediators of MEK/ERK1/2 and PI3K/AKT signaling pathways in TE5 and KYSE140 SCC cell lines following TP63, SOX2, or CCAT1 silencing. **f** Western blotting detection of phosphorylated EGFR (p-EGFR) and total EGFR in two SCC cell lines upon silencing of TP63, SOX2, or CCAT1. **g** Relative mRNA expression of EGFR upon knockdown of TP63, SOX2, or CCAT1 in TE5 and KYSE140 cells

To characterize the mechanisms underlying CCAT1-mediated cellular effects, RNA-Seq was first performed upon silencing of CCAT1 in different SCC cells. Interestingly, gene set enrichment analysis (GSEA) showed that super-enhancer-associated genes, but not typical-enhancer-associated genes, were preferentially downregulated upon silencing of CCAT1 (Fig. 5a), suggesting that silencing of CCAT1 disproportionately downregulated super-enhancer-mediated transcription. GO analysis revealed that these downregulated genes were enriched for processes involved in cancer biology, including cell proliferation, growth, and migration (Fig. 5b). This is in agreement with our findings that CCAT1 was required for SCC cell viability. Indeed, silencing of CCAT1 led to downregulation of many genes involved in cancer cell proliferation, survival and metastasis, such as *EGFR*, *CDK4*, *YES1*, *PAK4*, and *HMGA1* (Fig. 5c and Supplementary Data 9). Notably, both *EGFR* and *PAK4* were also associated with super-enhancers in SCC cells (Fig. 1a)^13^. Interestingly, genes involved in DNA and protein interactions (e.g., nucleotide binding, protein complex biogenesis and transcription cofactor binding) were also downregulated following CCAT1 knockdown (Fig. 5b), implying that CCAT1 might regulate the interaction of macromolecules.

To complement the investigations at RNA level, the Human Phospho-kinase array was utilized to detect changes of phosphorylated proteins associated with silencing of CCAT1. Notably, CCAT1 knockdown cells had reduced phosphorylation levels of a number of key molecules mediating the mitogen-activated protein kinase kinase/extracellular signal-regulated kinase 1/2 (MEK/ERK1/2) and phosphatidylinositol 3-kinase (PI3K/AKT) signaling pathways, including AKT, MEK1/2, ERK1/2, and p38 (MAPK) (Fig. 5d). Moreover, both upstream (EGFR) and downstream proteins (β-catenin, AMPK, WNK, and CREB) of these two pathways exhibited concordant reductions in their phosphorylation levels. Given the critical roles of these pathways in cancer biology, western blotting was performed to validate these changes. Indeed, the phosphorylation levels of all of the tested signaling molecules were consistently reduced in CCAT1-silenced SCC cells and xenografts compared to controls (Fig. 5e and Supplementary Fig. 1,0). Importantly, all of these reductions were also evident in either TP63 or SOX2 silenced cells (Fig. 5e), strongly supporting our earlier findings that CCAT1 was the downstream target of these two master TFs.

Integration of the results of both RNA-seq analysis and phospho-kinase array, drew attention to EGFR, a super-enhancer-associated oncogene, which exhibited consistent decreases in mRNA, protein and phosphorylation levels following CCAT1 depletion (Fig. 5f, g). Importantly, abundances of mRNA, total protein as well as the phosphorylation of EGFR were all markedly decreased upon silencing of either TP63, SOX2 or CCAT1 (Fig. 5f, g).

Moreover, the phosphorylation levels of key signaling mediators were restored upon overexpression of EGFR in CCAT1-silenced cells. In addition, overexpression of EGFR rescued significantly the decreased proliferation and colony growth of SCC cells induced by CCAT1 knockdown (Supplementary Fig. 1,1). These results characterized a novel regulatory cascade involving TP63/SOX2-CCAT1-EGFR, which activates both MEK/ERK1/2 and PI3K/AKT signaling pathways in SCC cells.

### CCAT1 recruits both TP63 and SOX2 to the super-enhancers of *EGFR* to promote its transcription

**Fig. 6:**
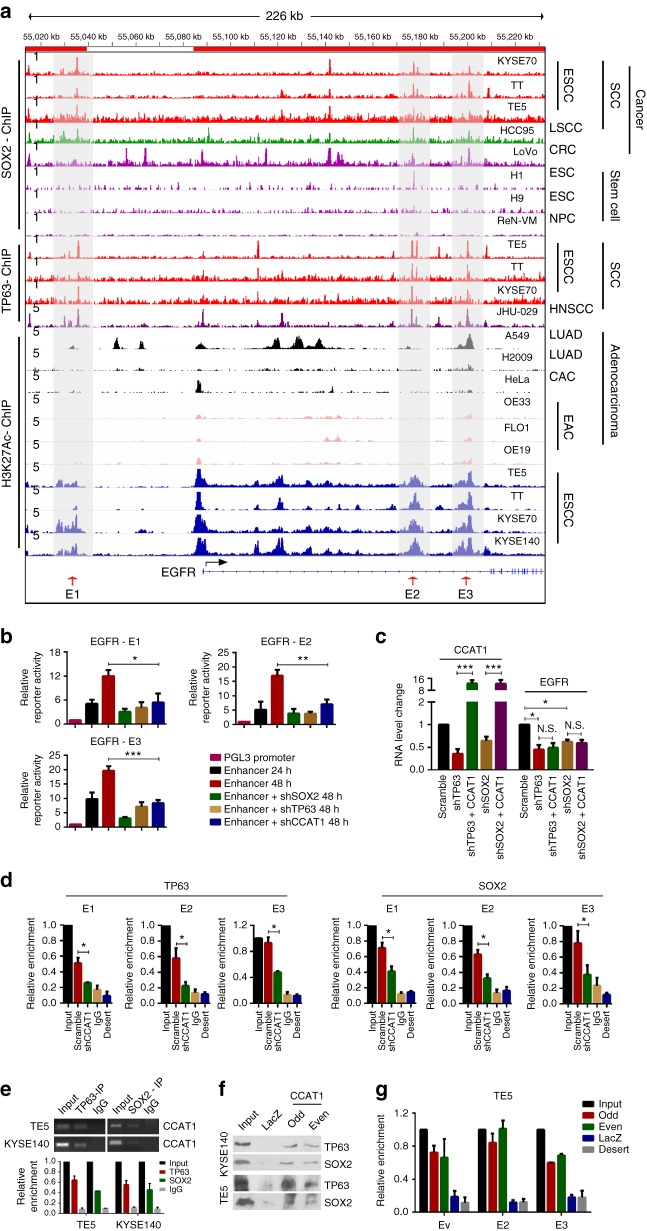
TP63/SOX2/CCAT1 form a complex and regulate EGFR transcription. **a** Gene tracks of SOX2, TP63, and H3K27ac ChIP-seq occupancy at super-enhancer domains (consist of E1, E2, and E3 enhancers) of *EGFR* in different types of SCC cells and adenocarcinoma cells. The grey shadings show SCCs-specific co-occupancy of SOX2, TP63, and H3K27ac at E1, E2, and E3 enhancer loci. **b** Relative luciferase activity upon transfection of each enhancer with or without silencing of TP63, SOX2, or CCAT1 in TE5 cells. **c** Expression of CCAT1 and EGFR upon silencing of TP63 or SOX2 and overexpression of CCAT1 (pcDNA3-CCAT1). **d** ChIP-qPCR experiments measuring TP63 and SOX2 binding enrichment on each *EGFR* enhancer segments upon silencing of CCAT1. Bars of **b**–**d** represent mean ± SD of three experimental replicates. * *P* < 0.05, ** *P* < 0.01, *** *P* < 0.001. *P* values were determined using *t*-test. **e** RNA immunoprecipitation (RIP) following by RT-PCR analysis detects the interaction between TP63/SOX2 and CCAT1 in TE5 and KYSE140 SCC cells. **f**, **g** ChIRP assay shows the interaction between CCAT1 and TP63 or SOX2. **f** ChIRP-immunoblotting with TP63, SOX2, or IgG antibody. **g** ChIRP-qPCR assays measure the enrichment of EGFR enhancer segments (E1, E2, and E3) in RNA-DNA complex. Probes against LacZ were used as a negative control. Desert was used as a negative control of genome locus. N.S.: not significant

To elucidate the mechanisms underlying TP63/SOX2-CCAT1-EGFR regulation, ChIP-seq data was initially interrogated. Notably, flanking *EGFR* were two super-enhancers (denoted by red bars on top of Fig. 6a), and TP63/SOX2 occupied both of them (shadowed regions containing E1, E2, and E3 enhancer) across all SCC cell lines (Fig. 6a). Again, these two super-enhancers were SCC-specific, being either not present or much weaker in LUAD, CAC and EAC cells. To evaluate the activity of these super-enhancer constituents, a series of luciferase reporter assay were performed. Each constituent (E1, E2, and E3) alone was capable of elevating the reporter activity (Fig. 6b and Supplementary Fig. 1,2). Importantly, all of these enhanced reporter activities were strongly reduced by silencing either TP63, SOX2, or CCAT1 (Fig. 6b and Supplementary Fig. 1,2), suggesting that the EGFR super-enhancers activity are modulated by TP63, SOX2, and CCAT1.

Rescue assays were next carried out to determine the mechanistic relationship between TP63/SOX2 and CCAT1 in the regulation of the *EGFR* super-enhancers. As expected, silencing of either TP63 or SOX2 reduced EGFR expression (Figs. 5g and 6c). Importantly, the reduction was not rescued by overexpression of CCAT1 (Fig. 6c and Supplementary Fig. 1,3), suggesting that CCAT1 cannot activate the *EGFR* enhancer independent of TP63 and SOX2. Notably, the abundance of occupancy of TP63 and SOX2 at super-enhancers (E1, E2 and E3) of *EGFR* substantially decreased after CCAT1 knockdown (Fig. 6d and Supplementary Fig. 1,4). These data suggest that the co-binding affinity of TP63/SOX2 at *EGFR* super-enhancers was maintained or reinforced by CCAT1 RNA. However, downregulation of CCAT1 did not affect the binding between TP63 and SOX2, suggesting that this protein-protein interaction is independent of CCAT1 (Supplementary Fig. 1,5). Given the physical association between TP63 and SOX2, these results also imply that TP63, SOX2, and CCAT1 might form a complex at these enhancers.

This hypothesis was first tested by RNA immunoprecipitation (RIP) pulling down the TP63- or SOX2-containing complex using either TP63 or SOX2 antibody. RT-PCR results showed that CCAT1 RNA strongly associated with TP63 and SOX2, but not with IgG antibody (Fig. 6e). To complement the RIP assay, chromatin isolation by RNA purification (ChIRP) was conducted. We designed a total of 13 probes tiling the full-length of CCAT1 RNA, and they were separated into odd and even pools^31,32^. Following IP, CCAT1-bound proteins and DNAs were detected by western blotting and qPCR assays, respectively. CCAT1 probes successfully retrieved ~60% of cellular CCAT1 RNA but minimal GAPDH RNA (negative control) (Supplementary Fig. 16a). On the other hand, probes tiling the Laz gene did not retrieve either CCAT1 or GAPDH RNA (Supplementary Fig. 16a). These results validated both the specificity and efficiency of CCAT1 ChIRP probes. Importantly, the interactions between CCAT1 RNA with both TP63 and SOX2 proteins were detected by western blotting in two different SCC cell lines (Fig. 6f), supporting the existence of a complex containing CCAT1 RNA, TP63, and SOX2 proteins.

We next examined DNA molecules, which were immunoprecipitated by CCAT1 ChIRP probes. Importantly, the *EGFR* super-enhancer constituents (E1, E2 and E3) were strongly enriched in the CCAT1-containing complex (Fig. 6g, Supplementary Figs. 1, 6b and 1, 6c). Moreover, knockdown of either TP63 or SOX2 markedly reduced the occupancy of CCAT1 in all three enhancer regions in comparison with control shRNA group (Supplementary Fig. 1,7). Taken together, the data demonstrate that a protein/nucleotide complex containing TP63/SOX2/CCAT1 occupies the super-enhancers of *EGFR* and promotes the transcription of EGFR in SCC cells.

## Discussion

TP63 and SOX2 are master TFs in SCC cells, which are also associated with super-enhancers themselves. However, whether and how super-enhancers are regulated by TP63 and SOX2 remains to be elucidated. In this study, we first noted that co-localization of TP63/SOX2 at super-enhancers occurred more frequently than unique occupancy by either TFs. Furthermore, this enrichment of co-binding was unique to super-enhancers, since it was not observed in typical-enhancer elements. Importantly, transcripts co-occupied by both TFs had higher expression relative to those bound by either TFs, suggesting stronger functional cooperation of TP63/SOX2 at super-enhancer elements.

Integrative analysis of both RNA-seq and ChIP-seq identified 52 transcripts as super-enhancer transcriptional targets directly co-occupied by both TP63 and SOX2. Among them, *CCAT1* and *TXNRD1* showed consistent changes across all three SCC cell lines examined. CCAT1 was detected to be distributed both in the nucleus and cytoplasm^29,33^. Previous reports studying gastric, colon and live cancers demonstrated that c-MYC could directly bind to the promoter region of *CCAT1* to enhance expression of this lncRNA, facilitating tumor progression^34–36^. Here, we observed that TP63 and SOX2 co-bound to the promoter and super-enhancer regions of *CCAT1* and contributed to its transcription activity specifically in SCCs, but not other cancer types. These results indicate tissue-specific regulations of CCAT1 transcription, which is consistent with the lineage-specific nature of TP63 in SCC cells.

Functionally, our results characterized *CCAT1* as a prominent oncogenic lncRNA in all four types of SCCs tested. To decipher the mechanisms responsible for the actions of CCAT1, both RNA-seq and phospho-kinase array were performed which identified EGFR as an important target of CCAT1. Nuclear lncRNAs have been observed to be involved in chromatin interactions, transcriptional regulation, and RNA processing by binding with DNA, RNA, and TFs; cytoplasmic lncRNAs can modulate stability or translation of transcripts and influence cellular signaling cascades^37–41^. For example, HOTAIR directly binds androgen receptor (AR), promotes AR-dependent transcriptional network and drives castration-resistant prostate cancer^42^. Notably, in the regulation of neurogenesis, SOX2 was found to interact with lncRNA-*RMST*, forming a protein/RNA complex binding at the promoter regions of several neurogenic TFs, including *SP8*, *HEY1*, *NEUROG2*, and *DLX1*. SOX2 occupancy at these promoters was reduced upon RMST knockdown, resulting in downregulation of the expression of these neurogenic TFs^43^. Similarly, using ChIP-qPCR and ChIRP-qPCR, we showed that both CCAT1 and SOX2 co-occupied the super-enhancer regions of *EGFR* in SCCs cells, and that CCAT1 was required for SOX2 binding (Fig. 6d, g). To the best of our knowledge, lncRNAs interacting with TP63 have not been identified previously. Here we showed the interaction of CCAT1 with TP63 and, together with SOX2, they co-bound *EGFR* super-enhancers and promoted their activities.

In colorectal cancer cells, a study noted that CCAT1 was located within a super-enhancer and interacted with CTCF protein to maintain chromatin looping between the *MYC* promoter and its enhancers, resulting in elevated expression of MYC^33^. In SCC cells, the mechanism by which CCAT1 regulates EGFR transcription appears similar to a certain degree, i.e., forming a protein/DNA/RNA complex within cis-regulatory regions. These results highlight *CCAT1* as an oncogenic enhancer RNA (eRNA) with unique functions in SCC cells^44^, as it regulates transcription via interacting with both master TFs (TP63 and SOX2) and DNA molecules (super-enhancer loci of EGFR). Occupancy of TP63/SOX2/CCAT1 complex at *EGFR* super-enhancers induced high transcription of the gene, which activates both MEK/ERK1/2 and PI3K/AKT signaling pathways, driving SCC cells proliferation and survival^45,46^. Interestingly, in gastric cancer cells CCAT1 modulated cancer cell proliferation, migration and invasion also by regulating the ERK/MAPK pathway^47^, congruent with our findings in SCC cells.

**Fig. 7:**
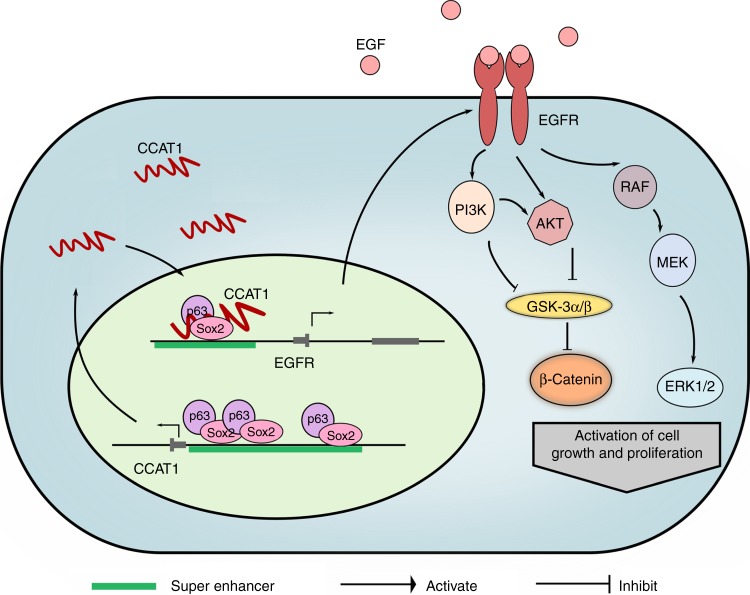
Proposed model of transcriptional dysregulation in SCC biology. TP63 and SOX2 co-bind at *CCAT1* promoter and super-enhancer regions, drive CCAT1 expression and form TP63/SOX2/CCAT1 complex, which occupies at super-enhancer domains of *EGFR*. This leads to the upregulation of EGFR expression, promoting SCC tumor development through activating MEK/ERK1/2 and PI3K/AKT signaling pathways

In summary, we provide compelling evidence to highlight a closely cooperative machinery between super-enhancers, lncRNA and master TFs that contribute to SCCs malignancy. SCC master TFs, TP63, and SOX2, transcriptionally activate CCAT1 RNA through direct co-occupying its promoter and super-enhancer elements. CCAT1 in turn recruits both TP63 and SOX2 and forms a protein/RNA complex, co-localizing at *EGFR* super-enhancers to activate its transcription. These complex regulations result in the high expression of both CCAT1 and EGFR, promoting SCC tumor progression through activating downstream MEK/ERK1/2 and PI3K/AKT signalings (Fig. 7).

## Methods

### Human cell lines

KYSE cell line series were provided by Dr Y Shimada (Kyoto University, Japan), and TE-5 and TT cells were provided by Dr Koji Kono (Cancer Science Institute of Singapore, Singapore). HNSCC cell lines (UMSCC1 and 93UV147) were kindly provided by Dr Timothy Chan (Memorial Sloan Kettering Cancer Center). LSCC cell lines (SK-MES-1, Calu-1, ChagoK1, H520, H2170 and H226) kindly provided by Dr GOH Boon Cher (Cancer Science Institute of Singapore). ESCC, LSCC, HNSCC, CSCC Cells were maintained at 37 °C in 5% CO2 in either RPMI-1640 medium or DMEM supplemented with 10% FBS, penicillin, and streptomycin. All these cells were recently authenticated by STR analysis^13,48^.

### Cell proliferation and colony formation assays

Cells were seeded into 96-well plates (2000–5000 cells/well) in quintuplicate. Cell proliferation was measured using MTT (3-(4, 5-dimethylthiazol-2-yl)-2, 5-diphenyl tetrazolium bromide) staining. For colony formation assay, cells were seeded into six-well plates (100–500 cells per well) and cultured for 2–3 weeks. Resulting colonies were calculated following 1% crystal violet staining.

### RNA extraction, cDNA synthesis, and quantitative PCR

Total RNA was extracted with RNeasy Mini kit (QIAGEN) and cDNA was obtained from the total RNA using EvoScript Universal cDNA Master (Roche). Quantitative PCR (qRT-PCR) was conducted with Precision^TM^FAST Mastermix (Precision, Precision-LR). GAPDH was used for normalization. Primers used in the study were listed in Supplementary Table 1.

### RNA-seq data analysis

The 100 bp paired end reads were aligned to hg19 Ensemble (V82) transcriptome using Kallisto pseudo aligner^49^. Transcript level abundances and counts were summarized to gene level using tximport Bioconductor package^50^. Differential gene expression analysis was performed using DESeq/DESeq2^51,52^. RNA-Seq data for KYSE70 and TT cell lines were downloaded from GEO (GSE47058) and processed in a similar way to avoid bias due to differences in analysis protocols. All differentially expressed genes (adjusted *P* value < 0.1) were used for Gene Ontology analysis using goseq Bioconductor package^53^. For GSEA analysis, we used all expressed genes with mean FPKM values > 0.5.

### Western blotting and IP

Cells were lysed with in RIPA lysis and extraction buffer (Thermo Fisher Scientific), supplemented with proteinase inhibitor cocktail, phosphatase inhibitor cocktail (Roche) for 30 min on ice. Protein concentrations were determined with Bio-Rad Protein Assay Kit (Bio-Rad) according to the instruction. Western blotting was performed using SDS-PAGE followed by transfer to nitrocellulose membrane (Bio-Rad). Primary antibody was incubated overnight in cold room. Secondary antibody was incubated for 1–2 h at room temperature.

For IP, 500 µg whole-cell lysate (for each experiment) was incubated with either indicated antibody or IgG on the rotary agitation overnight in cold room. The immunoprecipitates were then incubated with Dynabeads protein A/G (ThermoFisher Scientific) for 4 h in cold room, followed by purification and Western blotting analysis using indicated antibodies (Supplementary Fig. 1,5). Primary antibodies used were listed in Supplementary Table 2. Secondary antibodies were purchased from GE Healthcare SciMed.

### Plasmid transfection and lentiviral production

Lentiviral cloning vector pLKO.1-TRC (Plasmid #10878), pcDNA3.1-ΔNP63alpha-Flag (Plasmid # 26979), and pcDNA3.3-SOX2 (Plasmid #26817) plasmids were purchased from Addgene. The double-stranded oligonucleotide shRNAs were cloned into the AgeI/EcoRI sites of the pLKO.1-TRC lentiviral vector. Plasmid transfection, lentivirus production and lentivirus infection in cell lines were described previously^13,54^. shRNA target sequences were listed in Supplementary Table 3. As *TP63* has two isoforms-*TAp63* and ΔNp63, we designed two shRNAs, with shRNA-1 targeting all *TP63* isoforms and shRNA-2 specifically targeting only ΔNp63α isoform. Both of them were efficient in knocking down *TP63* mRNA and protein expression (Supplementary Fig. 4).

### Xenograft studies

Animal studies were performed in accordance with protocols approved by the ethical regulations of Institutional Animal Care and Use Committee (IACUC) of National University of Singapore. Xenograft models were established by subcutaneous injecting of 1 × 10^6^ TE5-Scramble or TE5-CCAT1 silencing cell lines (TE5-shCCAT1-1 or TE5-shCCAT1-2) into the flank of recipient NOD-SCID Gamma (NSG) mice (six weeks old, six mice). Mice general behaviors were monitored and the tumor volume was measured every 5 days for a total 20 days. At the end of the experiments, mice were sacrificed and the tumor tissues were collected for growth analysis.

### Luciferase reporter assay

Luciferase reporter vectors were purchased from Promega. The cDNA fragments of each promoter or enhancer regions containing were amplified using PCR and then inserted into pGL3-Promoter or pGL3-Enhancer vectors. The constructs with correct sequences were used for the transfection and a *Renilla* luciferase plasmid was co-transfected as a normalization control. The luciferase assays were carried out with Dual-Luciferase Reporter Assay System (Promega) and reporter activity was measured by Luminometer (Promega). The primers used for the amplification of each region were listed in Supplementary Table 4.

### Human phospho-kinase array

Human phospho-kinase array was performed according to the manual instructions (R & D Systems). Briefly, cell lysate were diluted into the desired quantity (1 µg/µl, 200–300 µg per sample for one experiment), incubated with membranes (A and B parts) overnight at 4 °C on a rocking platform. The membranes were then incubated with detection antibody cocktail A or B for 2 h following by Streptavidin-HRP for another 30 min at room temperature on a rocking platform shaker. Signal detection applied the Chemi Reagent Mix and exposed to film.

### RIP experiments

To study interactions of TP63 or SOX2 protein with CCAT1, RIP was performed using the published protocols^55^. Briefly, cell lysates were firstly incubated with Dynabeads protein A/G to remove non-specific binding. The precleared lysate was incubated with indicated antibody or the same type of IgG antibody at 4 °C overnight. Dynabeads were then added to immunoprecipitate antibody-antigen complexes at 4 °C for 4 h and the unbound proteins were washed off. RNA was then separated from the beads-antibody-RNA complexes for reverse transcription followed by PCR analysis.

### ChIRP analysis

ChIRP analysis was performed according to the published protocols^31,32^. CCAT1 anti-sense DNA probes with BiotinTEG at 3-prime end were designed using online probe designer (singlemoleculefish.com) and produced by Biosearch Technologies. Cells were collected and subjected to crosslink with 1% glutaraldehyde. The crosslinked cells were lysed and sheared into 100–500 bp DNA fragments by sonicating in a 4 °C water bath at highest setting with 30 s ON, 45 s OFF pulse intervals. CCAT1 biotinylated probes were separated into odd and even two pools to hybridize with RNA at 37 °C for 4 h with shaking. LacZ probes were used as a negative control. qRT-PCR was performed with RNA samples to confirm lncRNA-CCAT1 retrieval. Interaction of CCAT1 with DNA and proteins were examined by qPCR and Western blotting analysis. Probes used in this study are listed in Supplementary Table 5.

### ChIP sequencing and analysis

Cells were crosslinked with 1% formaldehyde solution and neutralized by 1.25 M glycine. Crosslinked cells were then lysed and sonicated with Bioruptor (Diagenode). Sonicated chromatin was precleared with Dynabeads protein A/G then incubated with indicated antibody for overnight at 4 °C and with beads for additional 2 h. DNA was eluted from immunoprecipitate complexes, reverse crosslinked and purified with QIAquick PCR spin kit (QIAGEN). High quality ChIP DNA were sequenced on Illumina HiSeq2000. ChIP-qPCR was performed to verify ChIP-seq results^13,54^. Gene desert (chr11: 127,277,673–127,322,674) serves as a negative control^56^. Primers for qPCR were shown in Supplementary Table 1. For ChIP-seq analysis, 50 bp single-end ChIP-seq reads were aligned to reference human genome (hg19) using bowtie short-read aligner, with alignment parameters –e70 –k2 –m2 –n2 –best –strata^57^. PCR duplicates were marked with picard MarkDuplicates and removed from further analysis. Peaks were identified using MACS2 peak caller with parameters –bdg –SPMR –nomodel –extsize 200 –q 0.01^58^. Bedgraph files generated with MACS2 were later converted bigwig using ucsc bedGraphToBigWig tool.

ChIP-seq data for TP63 and SOX2 in KYSE70 and TT cell lines were downloaded from GSE46837 and processed uniformly. TP63 and SOX2 peaks from all four cell lines were merged to generate a consensus peak sets. Overlapping SOX2 and TP63 peaks were considered as co-occupied regions. SOX2 or TP63 peaks with no nearby co-occupancy from the other TF within a distance of 1 kb were considered as unique peaks. In Fig. 1e, ChIP-seq signals from bigwig files were extracted using bwtool (summary and matrix subcommands) and manually plotted in R using base graphics or ComplexHeatmap Bioconductor package^59,60^. Super-enhancer-associated genes were identified using ROSE framework. All other test statistics were performed in R (VN. 3.3.0).

### Statistical analysis

For comparisons of continuous variables between groups, two-tailed Student *t* tests were used. Data were shown as the mean ± SD. The values at *P* < 0.05 (*), *P* < 0.01 (**), and *P* < 0.001 (***) were considered statistically significant. The details of statistical analysis were presented in figure legends. Diagrams were created by GraphPad Prism software.

## Electronic supplementary material

Supplementary Information

Description of Additional Supplementary Files

Supplementary Data 1

Supplementary Data 2

Supplementary Data 3

Supplementary Data 4

Supplementary Data 5

Supplementary Data 6

Supplementary Data 7

Supplementary Data 8

Supplementary Data 9

## Data Availability

The data supporting the findings of this study are available in the article or Supplementary Information files. The ChIP-seq and RNA-seq datasets have been deposited in the Gene Expression Omnibus (GEO) repository with the accession code GSE106563 and GSE106564.
