# Supplementary material for: Co-activation of super-enhancer-driven CCAT1 by TP63 and SOX2 promotes squamous cancer progression
Source: Nat Commun. 2018 Sep 6;9:3619. doi: 10.1038/s41467-018-06081-9 (PMC6127298; doi:10.1038/s41467-018-06081-9)
Supplement: Supplementary file 1 — Supplementary Information [file 41467_2018_6081_MOESM1_ESM.pdf]

Supplementary Information

Co-activation of super-enhancer-driven CCAT1 by TP63 and SOX2 promotes squamous cancer progression

Jiang et al.

Supplementary Figure 1

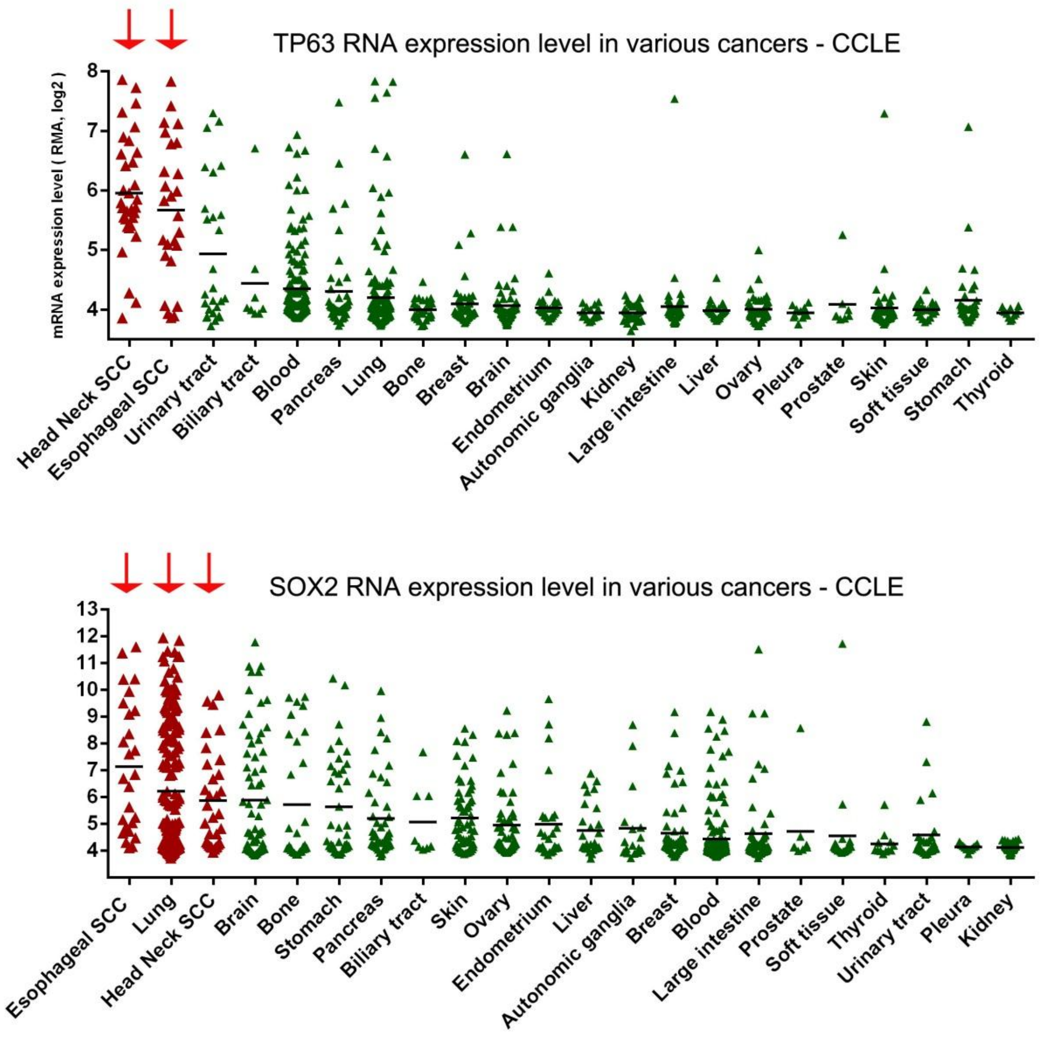

Supplementary Figure 1. Related to Figure 1. High expression of SCC master TFs TP63 and SOX2. mRNA expression levels of TP63 and SOX2 across various types of human cancers. Data were retrieved from the CCLE dataset. SCC cells were marked with red color and red arrows.

Supplementary Figure 2

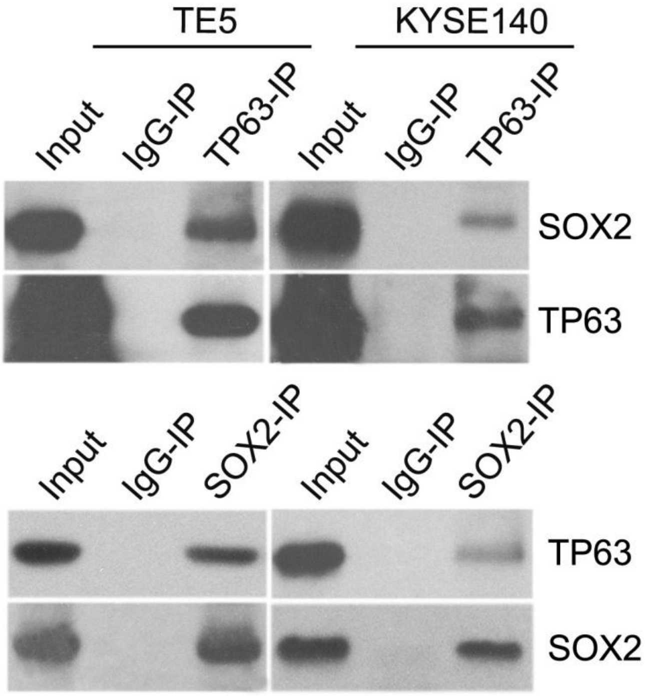

Supplementary Figure 2. Related to Figure 1. The interaction between TP63 and SOX2. Immunoprecipitation of TP63 or SOX2 using antibodies against endogenous SOX2 or TP63 in TE5 and KYSE140 cells.

Supplementary Figure 3

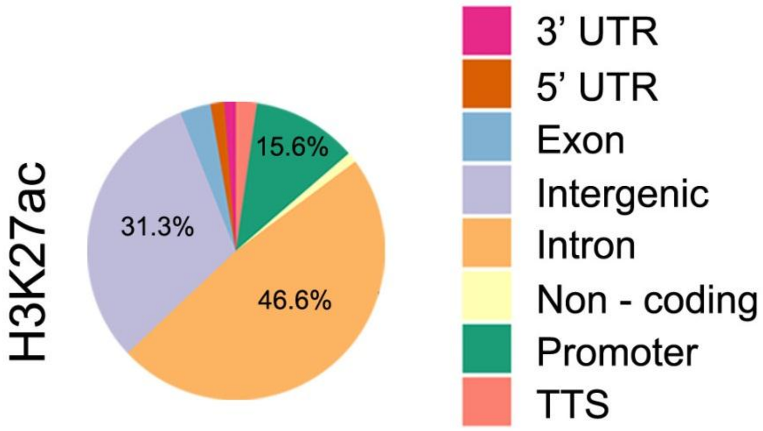

Supplementary Figure 3. Related to Figure 1. Genome-wide distribution of H3K27ac in ESCC cells.

Supplementary Figure 4

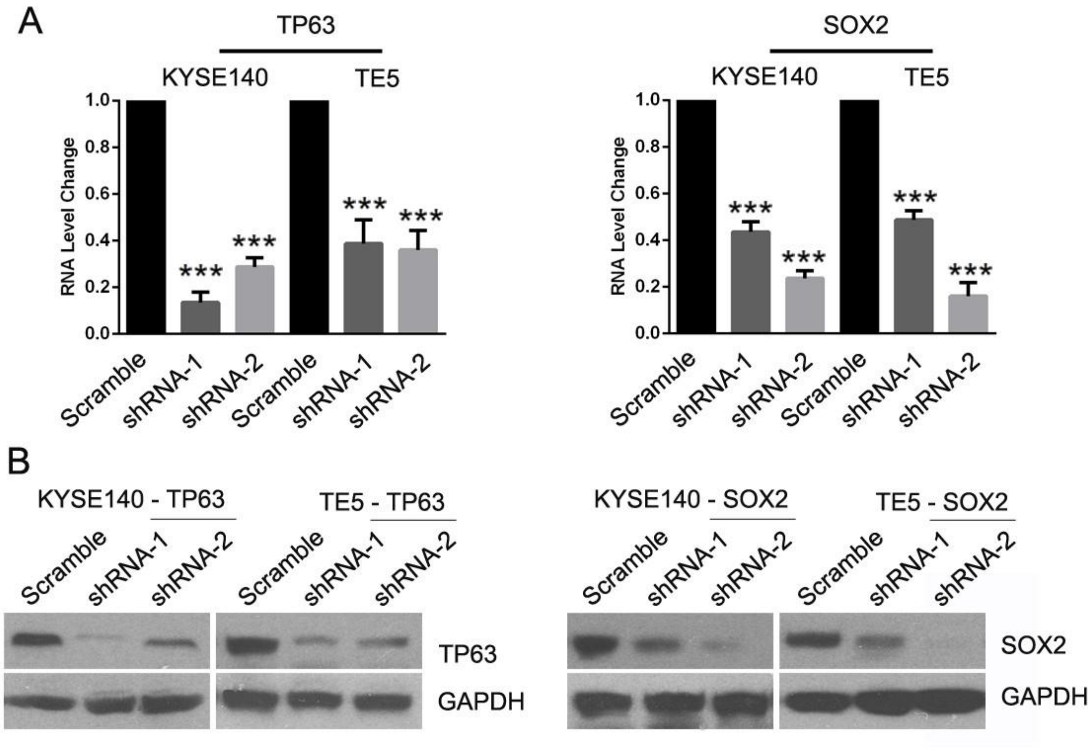

Supplementary Figure 4. Related to Figure 2. Silencing efficiency of TP63 and SOX2. (**a**) Relative mRNA expression upon silencing of TP63 or SOX2 with two shRNAs in KYSE140 and TE5 cells, measured by qRT-PCR (mean ± SD, n = 3). *** P < 0.001. (**b**) Efficient knockdown of TP63 and SOX2 in KYSE140 and TE5 cell lines, detected by western blotting using TP63 or SOX2 antibody respectively. GAPDH as a loading control.

Supplementary Figure 5

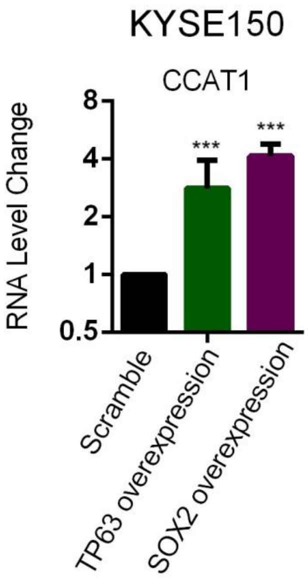

Supplementary Figure 5. Related to Figure 2. Forced expression of TP63 and SOX2 upregulate CCAT1 mRNA expression. Fold change of CCAT1 mRNA expression after overexpression of TP63 or SOX2 in KYSE140 and TE5 cells, measured by qRT-PCR (mean ± SD, n = 3). *** P < 0.001.

Supplementary Figure 6

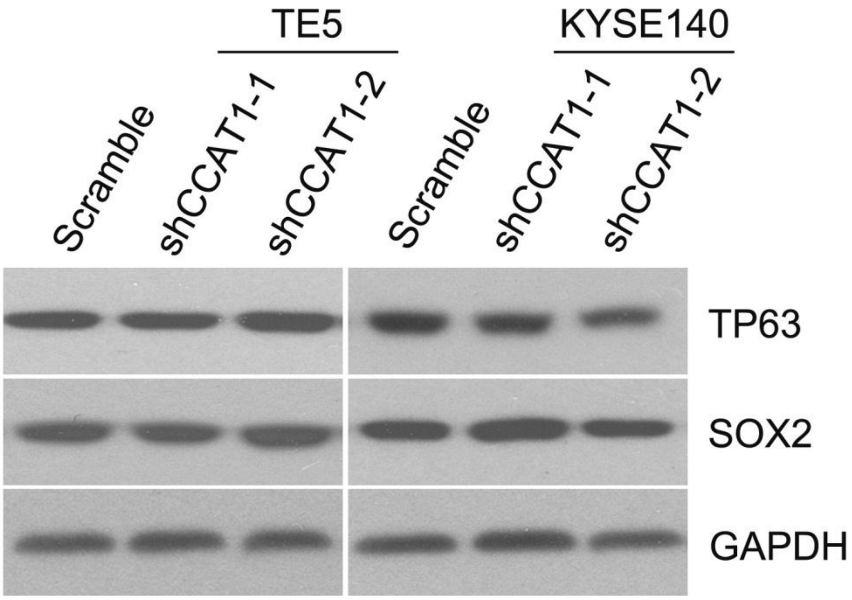

Supplementary Figure 6. Related to Figure 2. CCAT1 knockdown does not affect TP63 and SOX2 protein expression. Western blotting showing TP63 and SOX2 expression after silencing of CCAT1 with two shRNAs in TE5 and KYSE140 cells.

Supplementary Figure 7

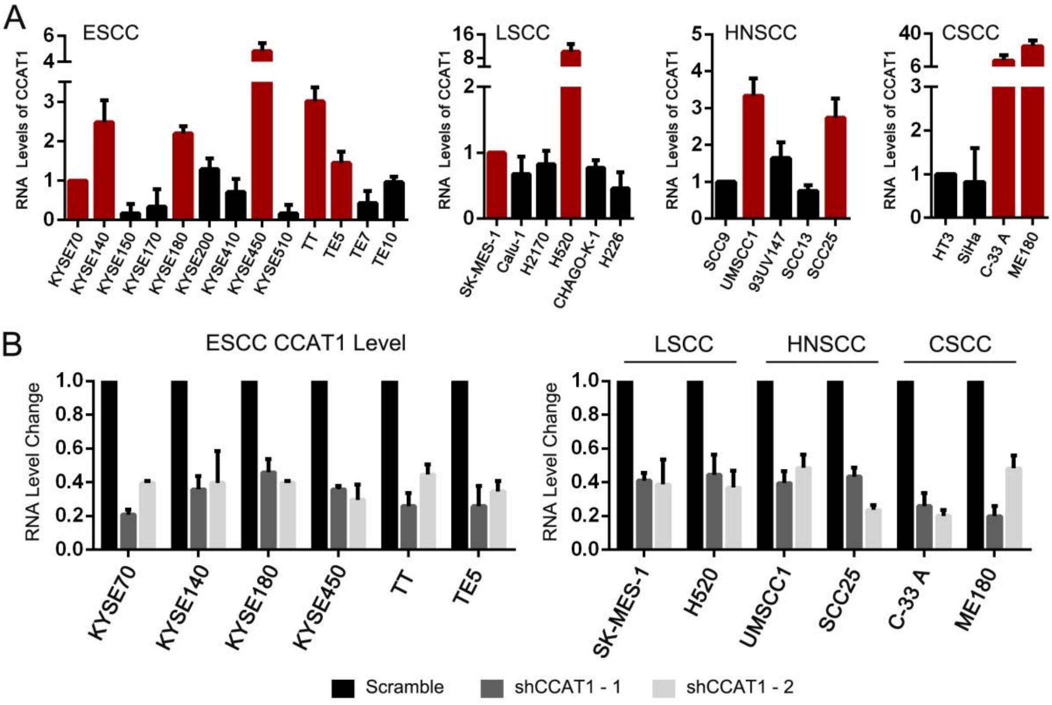

Supplementary Figure 7. Related to Figure 3. Knockdown efficiency of CCAT1. (**a**) Real-time PCR analysis showing the CCAT1 mRNA expression in 28 SCC cell lines, including ESCC, LSCC, HNSCC and cervical SCC (CSCC). The amount of mRNA was normalized to GAPDH levels. (**b**) mRNA level changes of CCAT1 upon shRNA-mediated silencing of CCAT1 in various of SCC cell lines, including ESCC, LSCC, HNSCC and CSCC.

Supplementary Figure 8

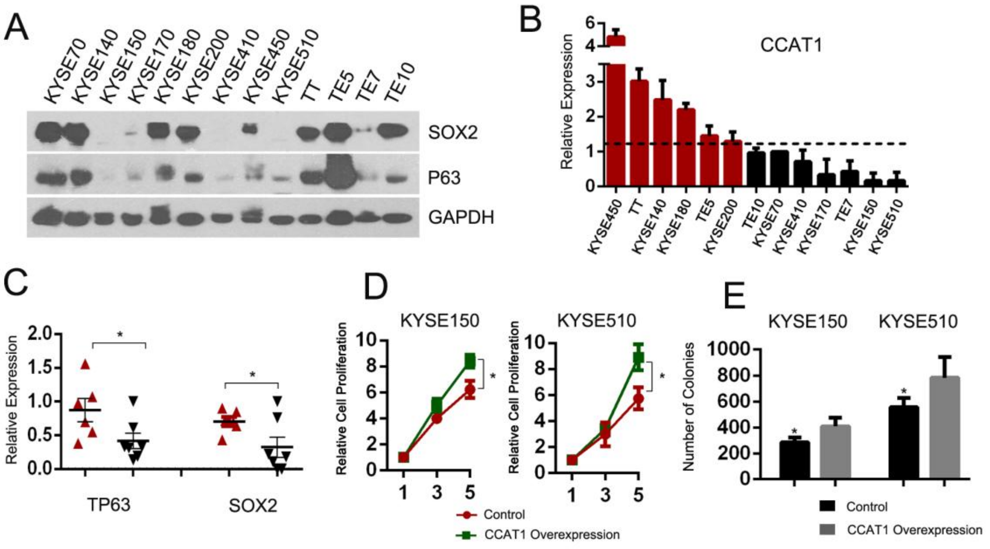

Supplementary Figure 8. Related to Figure 3. CCAT1 promotes the proliferation and viability of SCCs. (**a**) Immunoblotting analysis quantifying the protein levels of TP63 and SOX2 in 13 SCC cell lines. (**b**) Real-time PCR analysis measuring the RNA level of CCAT1 in the same 13 SCC cell lines, which were then divided into either CCAT1-high (Red) or CCAT1-low (Black) groups based on the median level of CCAT1 expression. (**c**) Dot plots showing the protein levels of TP63 and SOX2 in CCAT1-high (Red) and CCAT1-low (Black) groups. * $P < 0.05$. (**d**) Cell proliferation

and (**e**) colony formation assays in KYSE150 and KYSE510 cells upon CCAT1 overexpression.

Supplementary Figure 9

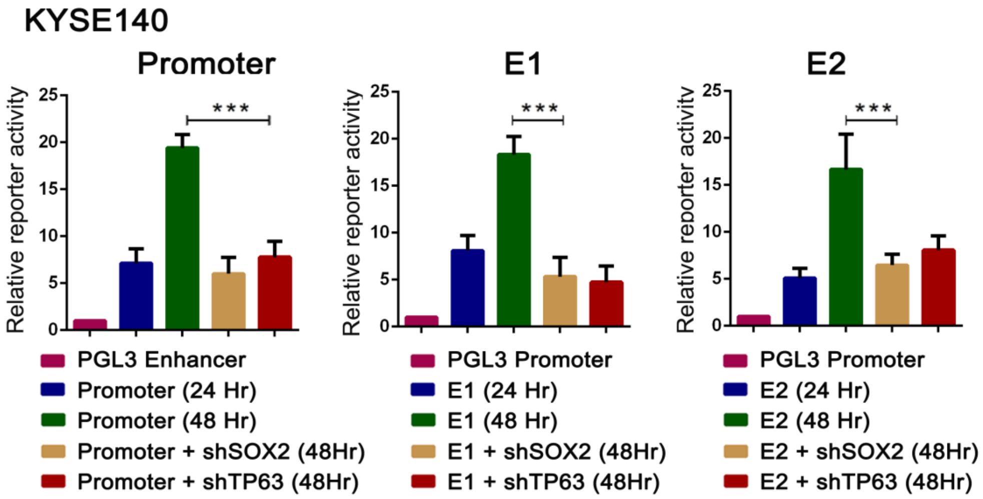

Supplementary Figure 9. Related to Figure 4. Luciferase reporter assay measuring the activity of *CCAT1* promoter and super-enhancer. Relative luciferase activity of CCAT1 promoter or enhancer reporter with or without TP63 or SOX2 silencing (shRNAs were transduced for 48 hours) in KYSE140 cells. Bars represent mean ± SD of three experimental replicates. *** $P < 0.001$.

Supplementary Figure 10

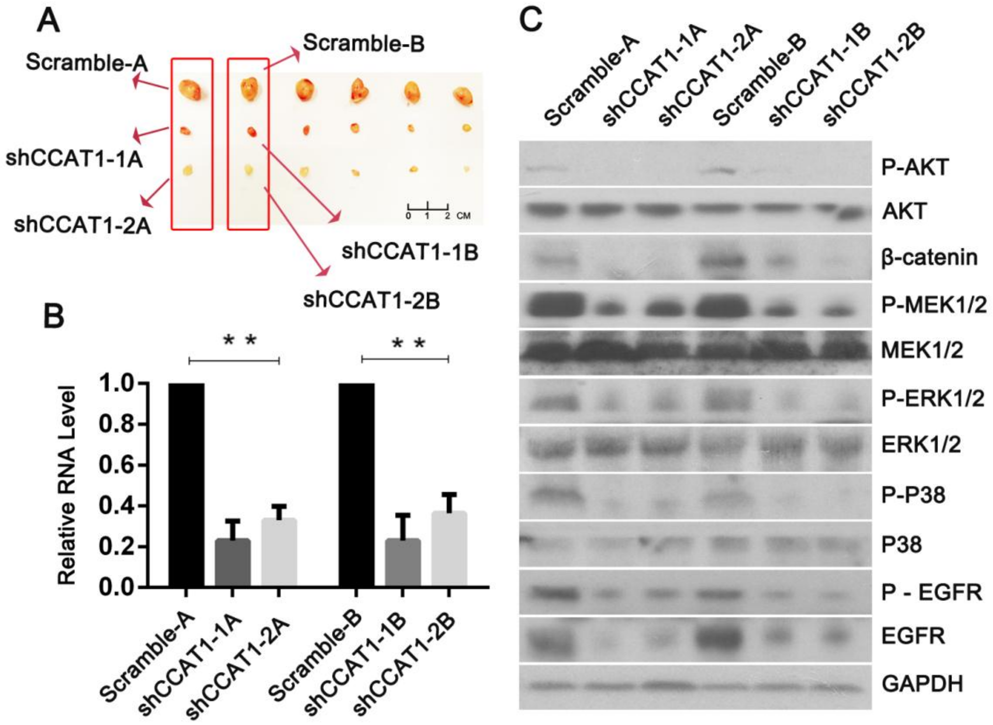

Supplementary Figure 10. Related to Figure 5. MEK/ERK1/2 and PI3K/AKT signaling pathways are regulated by CCAT1 *in vivo*. (**a**) Images of dissected tumors in control (Scramble) and two different CCAT1 knockdown groups (shCCAT1-1 and shCCAT1-2). Arrows indicate xenograft samples selected for molecular profiling. (**b**) Relative CCAT1 expression levels in xenografts from either control or two different CCAT1 knockdown groups, measured by qRT-PCR (mean ± SD, n = 3). ** $P < 0.01$. (**c**) Immunoblotting analysis measuring the levels of indicated proteins in xenografts from either control or two different CCAT1 knockdown groups.

Supplementary Figure 11

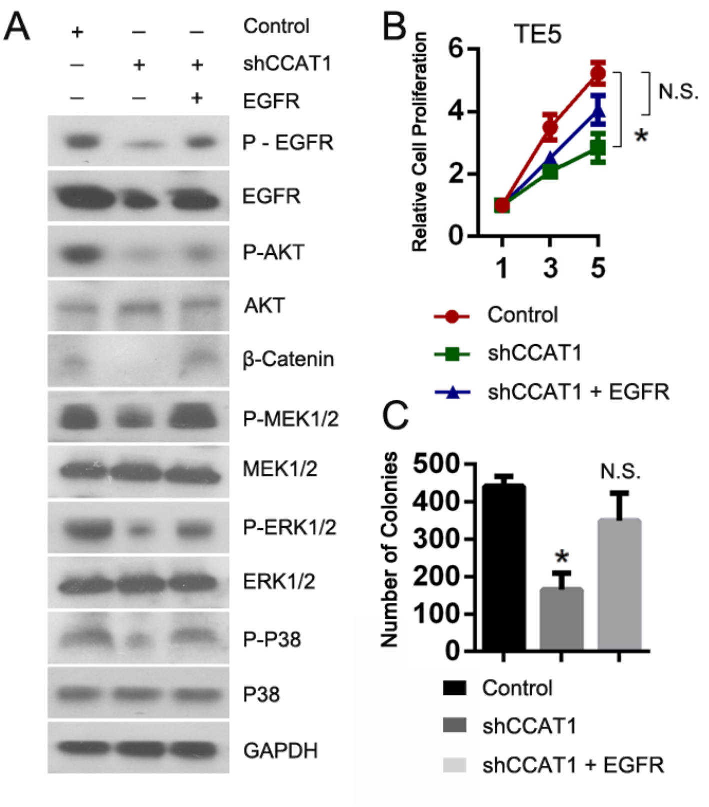

Supplementary Figure 11. Related to Figure 5. EGFR acts as a prominent functional mediator downstream of CCAT1. (**a**) Immunoblotting analysis measuring key signaling mediators of EGFR pathway following the overexpression of EGFR in CCAT1-silenced cells. (**b**) Cell proliferation and (**c**) colony formation assays of SCC cells following the overexpression of EGFR in CCAT1-silenced cells. * $P < 0.05$, ** $P < 0.01$, *** $P < 0.001$, N. S.: Not significant.

Supplementary Figure 12

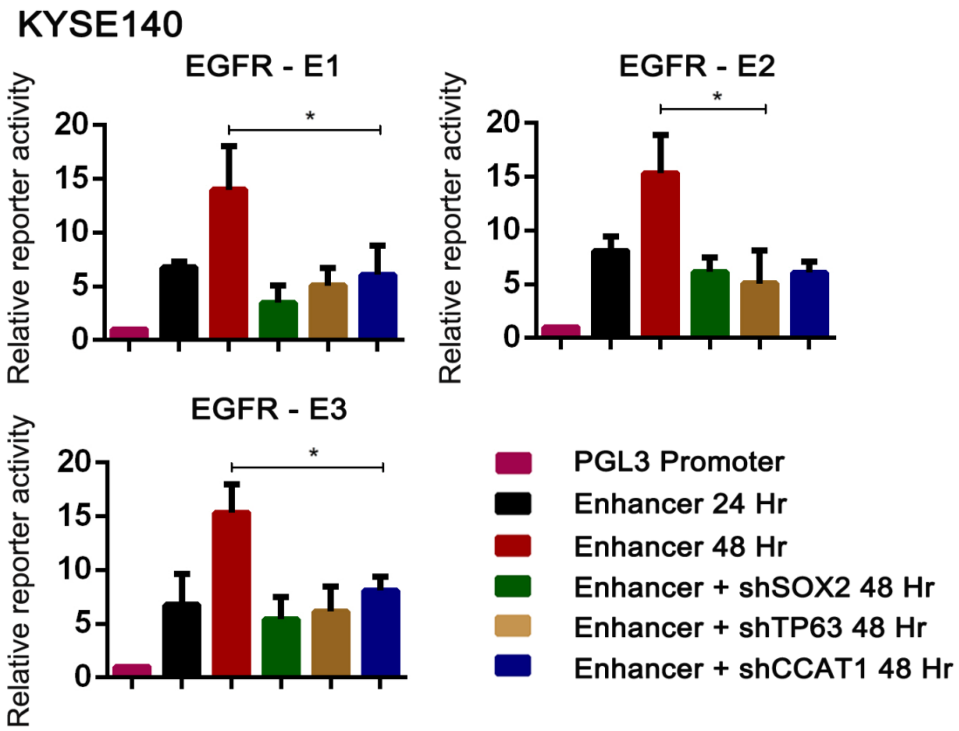

Supplementary Figure 12. Related to Figure 6. Luciferase reporter assay measuring *EGFR* super-enhancers activity. Each of the enhancer constituents (E1, E2 or E3) of *EGFR* super-enhancers showed high reporter activity which was reduced by shRNA-mediated knockdown of either TP63, SOX2 or CCAT1 in KYSE140 cells. Data represent mean ± SD of three experimental replicates. * $P < 0.05$.

Supplementary Figure 13

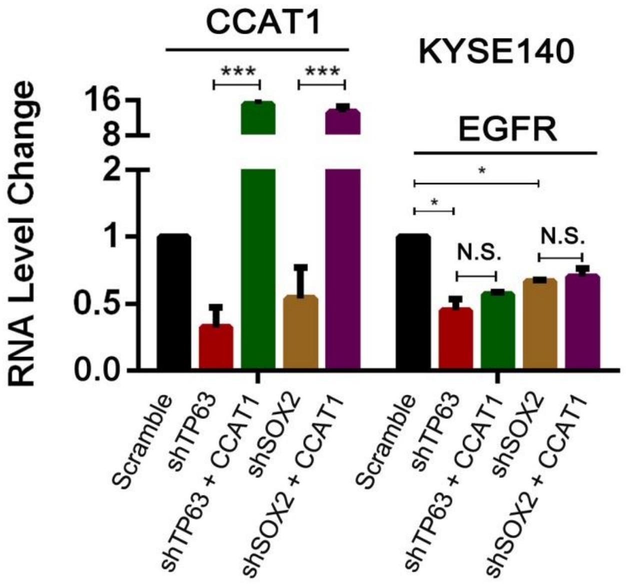

Supplementary Figure 13. Related to Figure 6. Expression of EGFR was regulated by TP63, SOX2 or CCAT1. Fold changes of mRNA expression for indicated genes after transfection of CCAT1 overexpression plasmid and shRNAs targeting TP63 or SOX2 in KYSE140 cells. Data showing with mean ± SD of three experimental replicates. * $P < 0.05$, *** $P < 0.001$. N. S.: Not significant.

Supplementary Figure 14

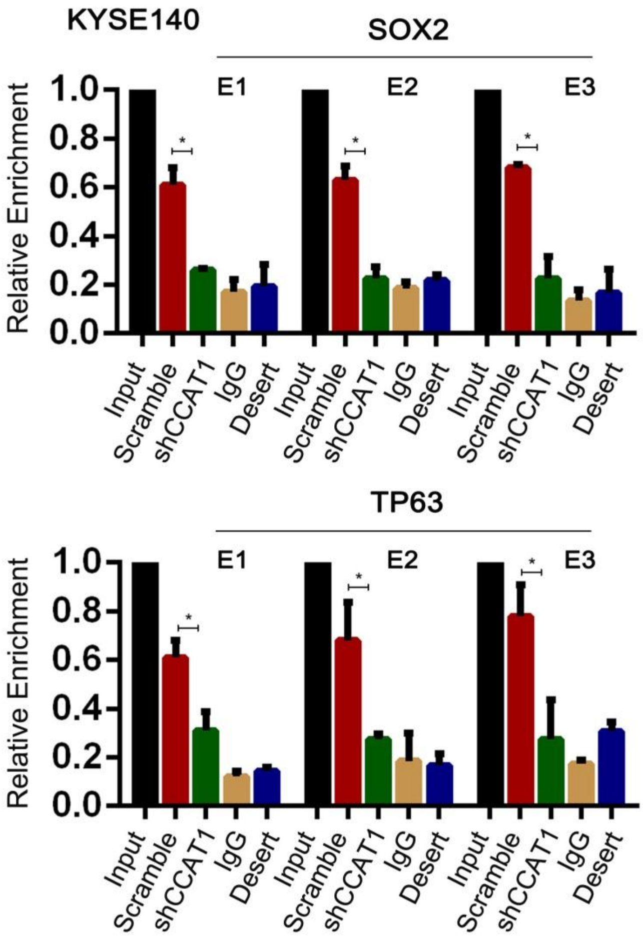

Supplementary Figure 14. Related to Figure 6. CCAT1 regulated TP63 and SOX2 occupancy at *EGFR* super-enhancer regions. Enrichment analysis of TP63 and SOX2 binding at *EGFR* super-enhancer region (E1, E2 and E3), measured by ChIP-qPCR in KYSE140 SCC cells. Silencing of CCAT1 inhibits such occupancy. Bars represent mean ± SD of three experimental replicates. * $P < 0.05$.

Supplementary Figure 15

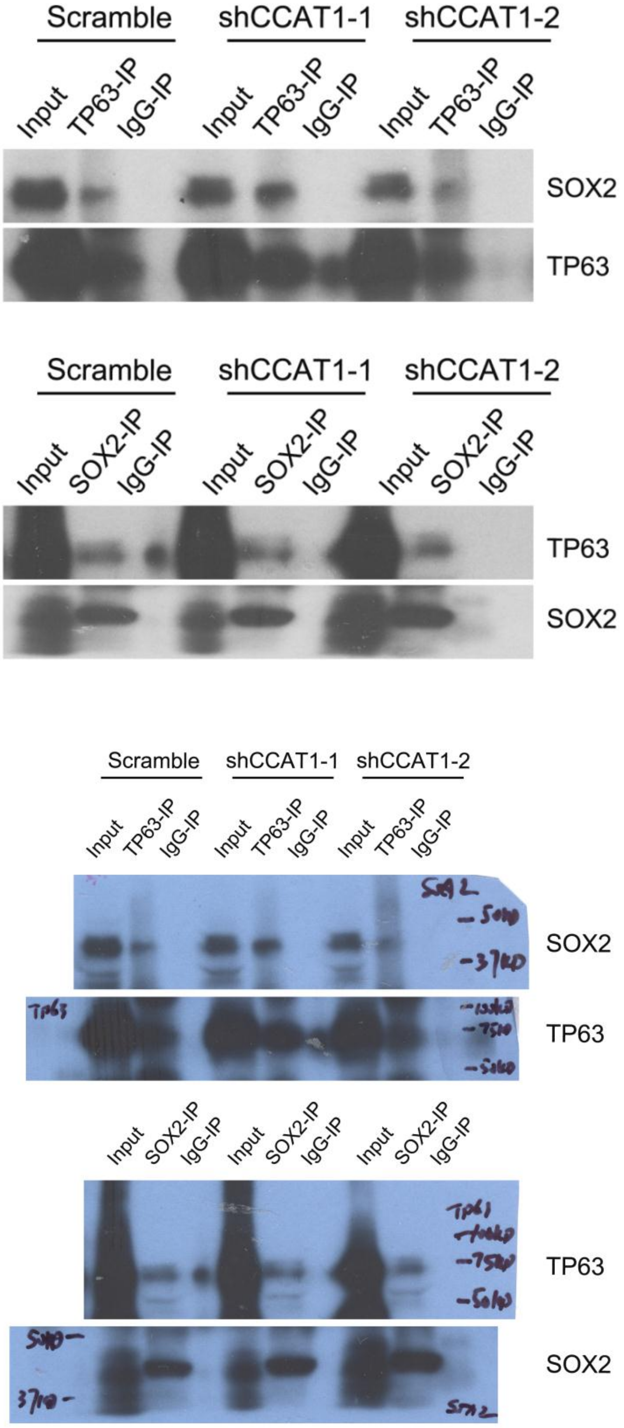

Supplementary Figure 15. Down-regulation of CCAT1 did not affect the binding between TP63 and SOX2.

Supplementary Figure 16

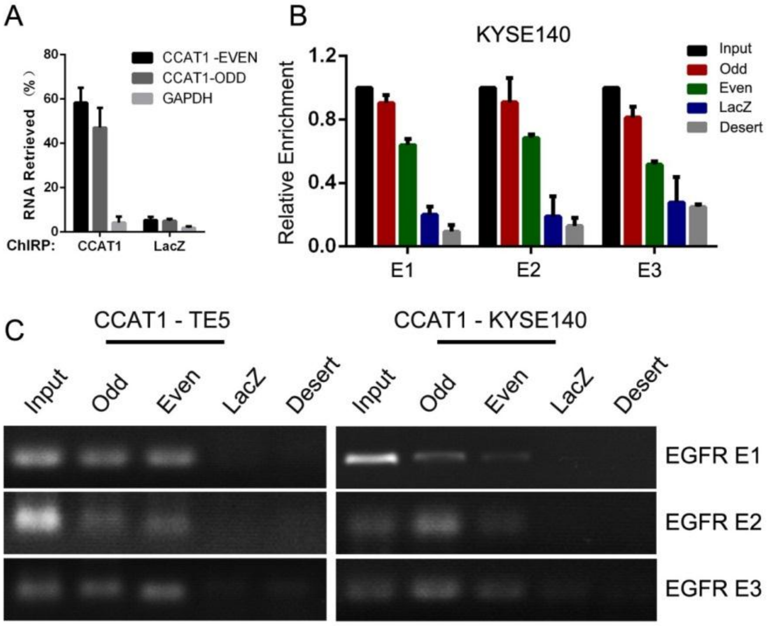

Supplementary Figure 16. Related to Figure 6. CCAT1 interacts with the super-enhancer regions of *EGFR*. (**a**) ChIRP followed by qRT-PCR analysis showed that CCAT1 probes were efficient to retrieve CCAT1 RNA in TE5 cells. LacZ probes were used as the negative control. (**b**) qPCR analysis and (**c**) gel electrophoresis analysis revealed that each enhancer (E1, E2 and E3) of *EGFR* super-enhancer was enriched in the immunoprecipitated RNA-DNA complex using odd or even probe pools in TE5 and KYSE140 cell lines. “LacZ” and “Dessert” served as the negative controls of probes and genomic loci, respectively.

Supplementary Figure 17

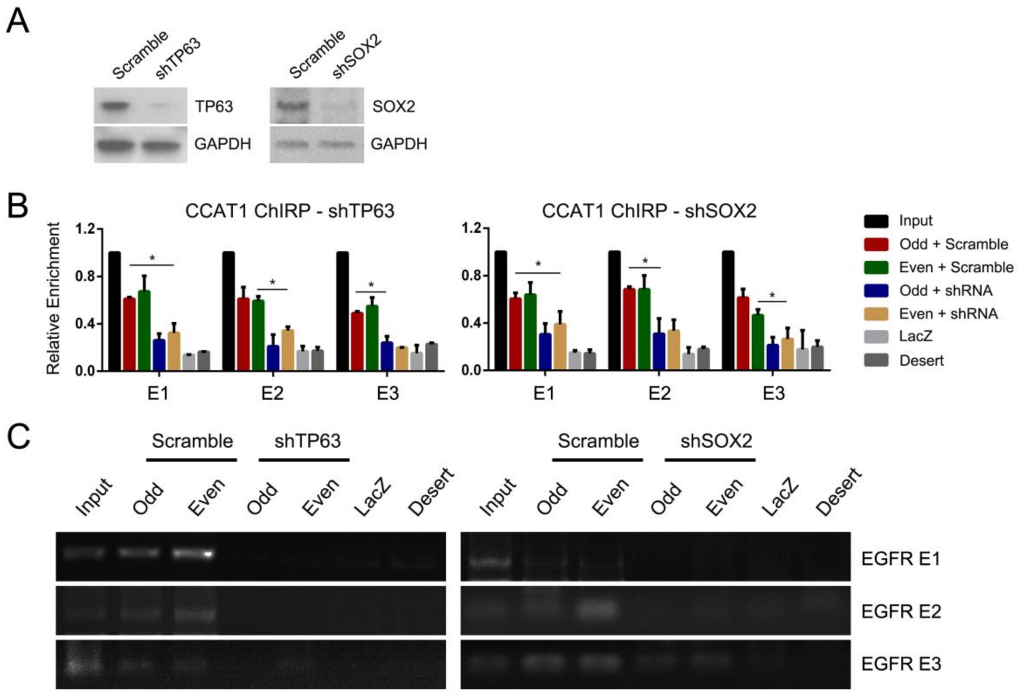

Supplementary Figure 17. TP63 or SOX2 depletion reduced the occupancy of CCAT1 in all three enhancer regions. (**a**) Immunoblotting assay validating knockdown efficiency of shRNAs against TP63 or SOX2. (**b**) ChIRP-qPCR assays measuring the enrichment of CCAT1 at EGFR super-enhancer segments (E1, E2 and E3) by different sets of CCAT1 ChIRP probes in the presence of either scramble shRNA or shRNAs targeting TP63 or SOX2. Probes tiling LacZ were used as a negative control of oligo probes. Gene desert was used as a negative control of genome locus. * $P < 0.05$. (**c**) Gel electrophoresis of ChIRP-PCR products showing the enrichment of CCAT1 by different sets of probes as defined in (**b**).

Supplementary Data 1. Related to Figure 1. Super-enhancer analysis of KYSE70 cells.

Provided as an excel file

Supplementary Data 2. Related to Figure 1. Super-enhancer analysis of KYSE140 cells.

Provided as an excel file

Supplementary Data 3. Related to Figure 1. Super-enhancer analysis of TE5 cells.

Provided as an excel file

Supplementary Data 4. Related to Figure 1. Super-enhancer analysis of TT cells.

Provided as an excel file

Supplementary Data 5. Related to Figure 2. Differentially expressed genes upon TP63 silencing in TE5 cells.

Provided as an excel file

Supplementary Data 6. Related to Figure 2. Differentially expressed genes upon SOX2 silencing in TE5 cells.

Provided as an excel file

Supplementary Data 7. Related to Figure 2. Differentially expressed genes upon TP63 silencing in KYSE140 cells.

Provided as an excel file

Supplementary Data 8. Related to Figure 2. Differentially expressed genes upon SOX2 silencing in KYSE140 cells.

Provided as an excel file

Supplementary Data 9. Related to Figure 5. Differentially expressed genes upon CCAT1 silencing in TE5 cells.

Provided as an excel file

Supplementary Table 1 | Oligonucleotide primers used for qPCR analysis.

	Forward	Reverse
TP63	GCCCCTCCTAGTCATTTGAT	ATCCCTCCAACACAACTGCT
SOX2	AGCTACAGCATGATGCAGGA	GGTCATGGAGTTGTACTGCA
CCAT1	ATTGGGAAAGGTGCCGAGAC	AGCGTCAGGGTTGACAGTAG
CCAT1-Promoter	CAGAGGTATGCGTAGGTGAT	ATCCCACTAGCAAGGACCAT
CCAT1-E1	TCATTCAGAAGCACCCAGAT	CACTGGGGCTAGAATTATGC
CCAT1-E2	GACAGTCATACAAGAGAGCA	CACAAGGAAGTATACAGACC

EGFR	TTTGCTGATTCAGGCTTGG	AGAAAACTGACCATGTTGCTTG
EGFR-E1	GTATCCAGAGTTCCCCACACG	GGCCTGAGAACATGAGGTGG
EGFR-E2	TGCTTACTGGTTAGGGCAGC	ACTCACATGGGAAAGCGAGG
EGFR-E3	GTGTGTGGACTGGGTGAGAC	TCCTAGACAGGCCCTCACAA
Desert	CCCTTGTAAGTTGGATTCCT	CAGCAAAGTCTCAGGATACA
GAPDH	GAAGGTGAAGGTCGGAGTC	GAAGATGGTGATGGGATTTC

Supplementary Table 2 | Antibodies.

TP63	sc-8431x<br>WB:1: 500<br>ChIP: 6ug
SOX2	AF2018 (R&D System)<br>WB:1ug/ml<br>ChIP: 6ug<br>3579S (Cell Signaling Technology)<br>1:1000

H3K27 acylation	ab4729 (Abcam) (3 – 6 ug)
Phospho-AKT	4060 (Cell Signaling Technology)<br>1:1000
AKT	4691S (Cell Signaling Technology)<br>1:1000
β-Catenin	9562S (Cell Signaling Technology)<br>1:1000
Phospho-MEK1/2	9154 (Cell Signaling Technology)<br>1:1000
MEK1/2	9122 (Cell Signaling Technology)<br>1:1000
Phospho-ERK1/2	9101 (Cell Signaling Technology)<br>1:1000
ERK1/2	9194 (Cell Signaling Technology)<br>1:1000
Phospho-P38	9216S (Cell Signaling Technology)<br>1:1000
P38	9212 (Cell Signaling Technology)<br>1:1000
Phospho-EGFR	2234S (Cell Signaling Technology)<br>1:1000
EGFR	4267 (Cell Signaling Technology)

	1:1000
GAPDH	2118 (Cell Signaling Technology)<br>1:5000

Supplementary Table 3 | shRNA target sequences.

Name	Target sequence
TP63-shRNA-1<br>(targets all the isoforms of TP63)	CCGTTTCGTCAGAACACACAT
TP63-shRNA-2<br>(specially targets ΔNp63)	GGACAGCAGCATTGATCAA
SOX2-shRNA-1	CGCTCATGAAGAAGGATAAGT
SOX2-shRNA-2	CTGCCGAGAATCCATGTATAT
CCAT1-shRNA-1	GCTTGGGACCACTGCTTTA
CCAT1-shRNA-2	CCATTCCATTCATTTCTCTTTCCTA
CCAT1-shRNA-3	CATTACCAGCTGCCGTGTTAA
CCAT1-shRNA-4	CGGATGGACATCAGAACTATT
CCAT1-shRNA-5	GTGAGTGAGATCAGCGTTATT

Supplementary Table 4 | Oligonucleotide primers used for PCR amplification of luciferase reporter vectors construction.

Name	Forward	Reverse
CCAT1-promoter	GGTACCTGTTGTGCCTAG	CTCGAGTTCAGGATCATC

	CAAGTGTA	ATTCTTAA
CCAT1-E1	GGTACCTGTTAGGATCTG AACCCATC	CTCGAGCCAAGTTCCACG TTCTGTGC
CCAT1-E2	GGTACCAGTCCTTCTAGC TGACTCCC	CTCGAGTTATGTATCAGA CATATAGA
EGFR-E1	GGTACCAGTGAAGGTTT ATGTGCTGCTT	CTCGAGTCAAGAACCACC ACTGCTCC
EGFR-E2	GGTACCTAAGCACCTCA ACACAGGCT	CTCGAGGAGGGAACCCA CAGCAAATG
EGFR-E3	GGTACCATTGGTGCTTTC GTCCCAGT	GCTAGCCGTGGGGATGAC GGAGATTG

Supplementary Table 5 | Anti-sense DNA probes used for ChIRP.

CCAT1-1	CTACAAGGCTCGGTTCAATT
CCAT1-2	GTTCGTTTACTTAACAGGGC
CCAT1-3	TTAAGGCTTGAATGCCTTCA
CCAT1-4	CGATGTTGTCAGTGAATGCT
CCAT1-5	TCTTCTTATGGTCTTCTCAC
CCAT1-6	CATCCGGTGTTTTTTCAGAA
CCAT1-7	GGAGTAGATTGGGTGATTCT
CCAT1-8	CCATCTTCTCAATGCCAAAG
CCAT1-9	TAACAGTTCCTATTACACCC

CCAT1-10	GGCTCAAGCATAAATGGGAC
CCAT1-11	TTGGGGTTTGGTCCACAATT
CCAT1-12	TACACACCTTCTTTCTCATC
CCAT1-13	CAAAGTATTCTAGCGAGCCT
LacZ-1	CGCGTAAAAATGCGCTCAGG
LacZ-2	GAGACGTCACGGAAAATGCC
LacZ-3	CACATCTGAACTTCAGCCTC
LacZ-4	TCATCGATAATTTCACCGCC
LacZ-5	TTCAACCACCGCACGATAGA
LacZ-6	CTCGAATCAGCAACGGCTTG
LacZ-7	GCGTTAAAGTTGTTCTGCTT
LacZ-8	ATGCCGTGGGTTTCAATATT
LacZ-9	TCGGCAAAGACCAGACCGTT
LacZ-10	CGCTATGACGGAACAGGTAT
LacZ-11	GTAGTTCAGGCAGTTCAATC
LacZ-12	AGCGTCACACTGAGGTTTTC
LacZ-13	AAGCCTGACTGGCGGTTAAA
LacZ-14	CTTCACTTACGCCAATGTCG
LacZ-15	CGCATCAGCAAGTGTATCTG
LacZ-16	CAACGGTAATCGCCATTTGA
