# Supplementary material for: Co-activation of super-enhancer-driven CCAT1 by TP63 and SOX2 promotes squamous cancer progression
Source: Nat Commun. 2018 Sep 6;9:3619. doi: 10.1038/s41467-018-06081-9 (PMC6127298; doi:10.1038/s41467-018-06081-9)
Supplement: Supplementary file 2 — Description of Additional Supplementary Files [file 41467_2018_6081_MOESM2_ESM.pdf]

Description of Additional Supplementary Files

File Name: Supplementary Data 1
Description: Super-Enhancer Analysis of KYSE70 Cells

File Name: Supplementary Data 2
Description: Super-Enhancer Analysis of KYSE140 Cells

File Name: Supplementary Data 3
Description: Super-Enhancer Analysis of TE5 Cells

File Name: Supplementary Data 4
Description: Super-Enhancer Analysis of TT Cells

File Name: Supplementary Data 5
Description: Expression Values per Transcript in Gene Expression Analysis of silencing TP63 in TE5 Cells

File Name: Supplementary Data 6
Description: Expression Values per Transcript in Gene Expression Analysis of silencing SOX2 in TE5 Cells

File Name: Supplementary Data 7
Description: Expression Values per Transcript in Gene Expression Analysis of silencing TP63 in KYSE140 Cells

File Name: Supplementary Data 8
Description: Expression Values per Transcript in Gene Expression Analysis of silencing SOX2 in KYSE140 Cells

File Name: Supplementary Data 9
Description: Expression Values per Transcript in Gene Expression Analysis of silencing CCAT1 in TE5 Cells
